# What can we learn from 100,000 freshwater forecasts? A synthesis from the NEON Ecological Forecasting Challenge

**DOI:** 10.1002/eap.70004

**Published:** 2025-02-12

**Authors:** Freya Olsson, Cayelan C. Carey, Carl Boettiger, Gregory Harrison, Robert Ladwig, Marcus F. Lapeyrolerie, Abigail S. L. Lewis, Mary E. Lofton, Felipe Montealegre‐Mora, Joseph S. Rabaey, Caleb J. Robbins, Xiao Yang, R. Quinn Thomas

**Affiliations:** ^1^ Department of Biological Sciences Virginia Tech Virginia USA; ^2^ Center for Ecosystem Forecasting, Virginia Tech Virginia USA; ^3^ Department of Environmental Science, Policy, and Management University of California Berkeley California USA; ^4^ Department of Ecoscience Aarhus University Aarhus Denmark; ^5^ Center for Limnology University of Wisconsin‐Madison Madison Wisconsin USA; ^6^ Large Lakes Observatory University of Minnesota Duluth Minnesota USA; ^7^ Institute of Arctic Biology University of Alaska Fairbanks Fairbanks Alaska USA; ^8^ Center for Reservoir and Aquatic Systems Research Baylor University Waco Texas USA; ^9^ Department of Earth Sciences Southern Methodist University Dallas Texas USA; ^10^ Department of Forest Resources and Environmental Conservation Virginia Tech Virginia USA

**Keywords:** ecological forecasting, forecasting challenge, freshwater, near‐term forecast, NEON, uncertainty, water quality

## Abstract

Near‐term, iterative ecological forecasts can be used to help understand and proactively manage ecosystems. To date, more forecasts have been developed for aquatic ecosystems than other ecosystems worldwide, likely motivated by the pressing need to conserve these essential and threatened ecosystems and increasing the availability of high‐frequency data. Forecasters have implemented many different modeling approaches to forecast freshwater variables, which have demonstrated promise at individual sites. However, a comprehensive analysis of the performance of varying forecast models across multiple sites is needed to understand broader controls on forecast performance. Forecasting challenges (i.e., community‐scale efforts to generate forecasts while also developing shared software, training materials, and best practices) present a useful platform for bridging this gap to evaluate how a range of modeling methods perform across axes of space, time, and ecological systems. Here, we analyzed forecasts from the aquatics theme of the National Ecological Observatory Network (NEON) Forecasting Challenge hosted by the Ecological Forecasting Initiative. Over 100,000 probabilistic forecasts of water temperature and dissolved oxygen concentration for 1–30 days ahead across seven NEON‐monitored lakes were submitted in 2023. We assessed how forecast performance varied among models with different structures, covariates, and sources of uncertainty relative to baseline null models. A similar proportion of forecast models were skillful across both variables (34%–40%), although more individual models outperformed the baseline models in forecasting water temperature (10 models out of 29) than dissolved oxygen (6 models out of 15). These top performing models came from a range of classes and structures. For water temperature, we found that forecast skill degraded with increases in forecast horizons, process‐based models, and models that included air temperature as a covariate generally exhibited the highest forecast performance, and that the most skillful forecasts often accounted for more sources of uncertainty than the lower performing models. The most skillful forecasts were for sites where observations were most divergent from historical conditions (resulting in poor baseline model performance). Overall, the NEON Forecasting Challenge provides an exciting opportunity for a model intercomparison to learn about the relative strengths of a diverse suite of models and advance our understanding of freshwater ecosystem predictability.

## INTRODUCTION

Ecological forecasting is a growing field that leverages predictions of future ecological states to help understand and manage ecosystems (Dietze et al., 2018; Lewis et al., 2023; Tulloch et al., 2020). Here, we define forecasts as predictions of future conditions with specified uncertainty (Lewis et al., 2022). As environmental conditions increasingly change in response to altered climate and land use (Arias et al., 2021), ecological forecasts have considerable potential for improving management to support ecosystem services now and in the future (Bradford et al., 2018; Dietze et al., 2018). Moreover, forecasting future conditions that have yet to occur inherently requires out‐of‐sample implementation of models, which can lead to insights into optimal modeling approaches (Lewis et al., 2023).

In freshwater ecosystems, rapid environmental change has led to conditions that are both more variable and outside of historically observed states, motivating a particular need for near‐term, iterative ecological forecasts (e.g., Carey, 2023; Richardson et al., 2024; Siam & Eltahir, 2017). Near‐term (i.e., subdaily to decadal) forecasts allow researchers to evaluate models within management‐relevant timescales (Dietze et al., 2018), and iteratively updating and evaluating forecasts enables rapid improvement in forecast performance by integrating observational data and updating parameters (Dietze et al., 2018; Loescher et al., 2017). These near‐term iterative ecological forecasts will help protect critical provisioning, regulating, supporting, and cultural services (Dodds et al., 2013; Lofton et al., 2023; Sterner et al., 2020) that these highly threatened systems provide (Carrizo et al., 2017; Dudgeon et al., 2006; Reid et al., 2019), thereby improving management and mitigation (e.g., Carey et al., 2022; Huang et al., 2011; Zwart et al., 2023).

Although the number of near‐term, iterative water quality forecasts of freshwater ecosystems is growing (Lofton et al., 2023), challenges remain in producing reliable and accurate predictions of changes in these environments. To date, researchers have implemented many classes of models to forecast freshwater variables (reviewed by Lofton et al., 2023), including process‐based (PB) models (Baracchini et al., 2020; Clayer et al., 2023; Page et al., 2018; Thomas et al., 2020), machine learning (ML) models (Cheng et al., 2020; Di Nunno et al., 2023; Read et al., 2019; Zwart et al., 2023), statistical models (Caissie et al., 2017; McClure et al., 2021; Woelmer et al., 2021), and multimodel and hybrid approaches (Olsson, Moore, et al., 2024; Qu et al., 2017; Saber et al., 2020). In addition, forecasts have been generated using a range of model covariates (i.e., driver variables). In many cases, weather forecasts are used as covariates because meteorology is a key driver of many ecosystem processes in freshwater ecosystems (Hipsey et al., 2019; Livingstone & Padisák, 2007; Rousso et al., 2020). Additionally, some models include autoregressive terms as covariates (e.g., ARIMA models). While forecasting methods have demonstrated promise at individual freshwater sites or a handful of sites (e.g., Barrachini et al., 2020; Chen et al., 2024; Ouellet‐Proulx et al., 2017; Page et al., 2018; Thomas et al., 2020; Zwart et al., 2023), to date there has yet to be a comprehensive analysis of the performance of forecasting models across a large range of model classes and model covariates across multiple sites.

Forecasting challenges present a useful platform for bridging this gap and learning about how a range of modeling methods perform across axes of space, time, and ecological systems (Humphries et al., 2018; Thomas et al., 2023). Forecasting challenges typically entail an open call to the research community with a “challenge” to forecast a specific variable, standardized requirements, and formal evaluation of out‐of‐sample time steps. Some challenges have aimed to identify a “winner” or best approach, while others have focused more on community and knowledge building (Humphries et al., 2018; Makridakis et al., 2020; Thomas et al., 2023). By bringing together individuals and teams from broad backgrounds, challenges provide opportunities for innovation and community‐building, and the development of community cyberinfrastructure can accelerate discipline‐wide progress (Fer et al., 2021). Altogether, this collaborative effort can facilitate the development of new methods, standardization of forecasting targets and formats, and tools and templates that expand the training and education to improve accessibility of forecasting (Thomas et al., 2023). While forecasting challenges are common in the fields of finance, business, demography (Bojer & Meldgaard, 2021; Makridakis et al., 2020), and epidemiology (Biggerstaff et al., 2018; Johansson et al., 2019; Viboud et al., 2018), few have existed in ecology until recently (e.g., Humphries et al., 2018; Wheeler et al., 2024), providing new opportunities for advancing the discipline. For example, previous efforts to compare outcomes among ecological forecasting methods have been hindered by differences in evaluation metrics, sites, and variables being forecasted (e.g., Rousso et al., 2020), which can be addressed by a standardized forecasting challenge framework.

The National Ecological Observatory Network (NEON) Forecasting Challenge (hereafter NEON Challenge), hosted by the Ecological Forecasting Initiative (EFI) Research Coordination Network, was designed to initiate these advances in ecological forecasting. The NEON Challenge is “an open platform for the ecological and data science communities to forecast NEON data before they are collected” (Thomas et al., 2023). The challenge aims to galvanize the forecasting community around a common framework, with the goals of improving forecasting tools (e.g., Dietze et al., 2023), learning about ecological predictability (e.g., Wheeler et al., 2024), and advancing training (e.g., Willson et al., 2023).

The NEON Challenge provides a unique case study for examining the performance of freshwater forecasts across space, time, and ecological systems. Ecological time series present specific complexities compared with previous forecasting challenges given the variability in ecological data collection, irregularities in data resolution, and the inherent variability of the observations (Farley et al., 2018; Michener & Jones, 2012). Moreover, unlike previous forecasting challenges, the NEON Challenge is ongoing and accepts submissions of as‐yet‐unmeasured conditions on a rolling basis, with scoring occurring continuously as new data are collected and made available in near real time (Thomas et al., 2023). In the aquatics lake theme of the NEON Challenge, participants were invited to submit 1‐ to 30‐day‐ahead probabilistic forecasts of daily surface mean water temperature (hereafter, *T*_*w*_) and dissolved oxygen concentration (DO) of seven NEON lake sites, with new forecasts accepted daily (Thomas et al., 2023). Due to issues relating to data quality, submitted forecasts of chlorophyll *a* were omitted from our analysis. Forecasts were solicited across a range of sites, dates, and variables to understand how skill varies across these three axes. Forecasts could be generated using any method but had to include an estimate of uncertainty.

The inclusion of, and emphasis on, uncertainty was a novel component of the NEON Challenge, as uncertainty has been rarely included in previous forecasting challenges. Meaningful representations of uncertainty are critical to forecast interpretation and comparison, but uncertainty quantification is still not ubiquitous across ecological forecasts (reviewed by Lewis et al., 2022), and freshwater forecasts in particular. In a review of freshwater forecasts by Lofton et al. (2023), only 16 out of 61 near‐term (subdaily to decadal) forecasts of water quality variables included an estimate of the uncertainty associated with a prediction. Uncertainty can arise from a variety of sources: model process, model parameters, model initial conditions, model drivers, and observations (Table 1). The relative importance of each source is often dependent on the ecosystem process or state being forecasted and the forecast horizon (Lofton et al., 2022; Ouellet‐Proulx et al., 2017; Thomas et al., 2020). In addition, the predictability of an ecological process or state depends on the magnitude of the forecast spread (forecast uncertainty) and the rate at which uncertainty increases across the forecast horizon. Predictability is low when forecast spread is large enough that it cannot distinguish between consequential differences in ecosystem processes or states, or when it is no different from random chance. Forecast spread in turn depends on the sources of uncertainty in the forecast model (Dietze, 2017) and the model sensitivity to these sources. For example, the predictability of ecosystem processes that are sensitive to meteorological drivers (e.g., air temperature) depends on the uncertainty in the weather forecasts used as inputs to the ecological forecast model (Dietze, 2017).

**TABLE 1:** Definitions of forecast uncertainty sources included in the submitted models, modified from Dietze (2017), Lofton et al. (2023), and Thomas et al. (2020).

Source of uncertainty	Definition	Example of how the uncertainty source could be quantified
Process	Uncertainty from the inability of the model to replicate the dynamics of the forecasted state.	Calculating the error from the residuals of the model fit to historical data.
Parameter	Uncertainty in the parameter values of a fitted model.	Sampling from a distribution of parameter values and assigning different parameter values to each ensemble member.
Initial condition	Uncertainty in estimates of current conditions at the time of forecast generation (e.g., as a result of observation uncertainty, missing observations, and data assimilation).	Quantifying the spread in updated states following data assimilation or the previous day's forecast.
Driver	Uncertainty from driver data (e.g., future air temperature).	Using an ensemble of weather forecasts as drivers to the model.
Observation	Uncertainty from measurement error in the state being forecasted (difference between actual state and measured state).	Calculating the standard deviation of replicate water temperature observations.

We were specifically focused on uncertainty in our analysis because forecasts that include well‐quantified uncertainty, in addition to being accurate, have been shown to improve decision‐making outcomes (Mylne, 2002; Nadav‐Greenberg & Joslyn, 2009; Ramos et al., 2013). NEON forecast submissions were thus evaluated in two ways that captured different attributes of accuracy and precision: the continuous rank probability score (CRPS), a CRPS comparison with a baseline (null) model that acted as a benchmark to assess relative gains in forecast performance (forecast skill; Murphy, 1992; Pappenberger et al., 2015), and an evaluation of how well the forecast CIs capture the observation (CI reliability; e.g., if 90% of the observations in the 90% forecast CI).

In this study, we analyzed a year of submissions to the aquatics theme of the NEON Challenge and assessed how model performance varied among model class, model covariates, and forecast sites. We used the forecast analysis to answer the following research questions: Q1: How does model class and inclusion of covariates affect forecast performance? Q2: To what extent is relative forecast skill affected by the inclusion of different sources of uncertainty? Q3: How consistent are the patterns in forecast performance across sites? We included all *T*_*w*_ and DO forecasts in the analysis of Q1 but focused primarily on *T*_*w*_ forecasts for Q2 and Q3 due to the much higher number of submissions for that variable (see below). To the best of our knowledge, our study is the first analysis that investigates the performance of freshwater forecasts across multiple model classes, model covariates, and sites using genuine forecasts of the future.

## METHODS

### NEON challenge overview

The NEON Challenge has five forecasting themes that cover a range of ecological populations, communities, and ecosystems across the NEON network of monitored freshwater and terrestrial sites. Our coauthor team represents a group of the Challenge organizers, cyberinfrastructure developers, and/or forecast submitters.

Submissions were accepted to the aquatics theme of the NEON Challenge starting in 2021 and continuing to the present (>3 years) for forecasts of water quality. Here, we focus on the forecasts of *T*_*w*_ and DO submitted to lake sites within the aquatics theme of the NEON Challenge during 2023, which represented the first full year with sufficient submissions for a robust intermodel comparison.

### Challenge design

#### NEON data

**FIGURE 1:**
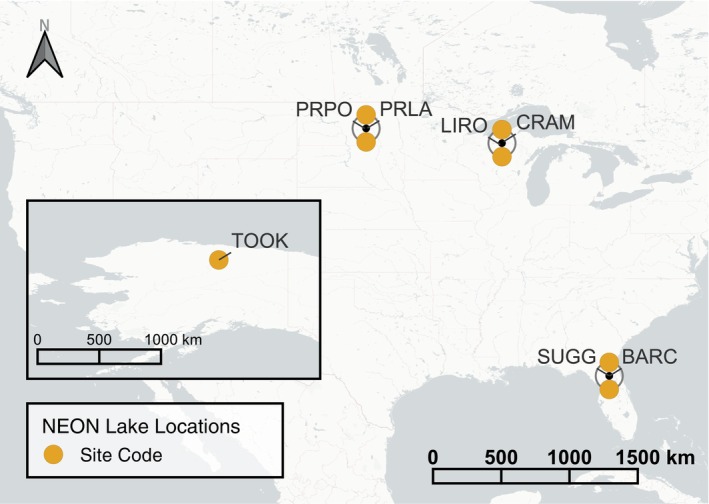
Map of National Ecological Observatory Network (NEON) lake sites located across the contiguous United States, with map inset showing Alaska. Co‐occurring sites are shown by the black centroid and the colored points are offset from this location. The points are labeled with their four‐character NEON site code: BARC, Barco Lake; CRAM, Crampton Lake; LIRO, Little Rock Lake; PRLA, Prairie Lake; PRPO, Prairie Pothole Lake; SUGG, Suggs Lake; TOOK, Toolik Lake.

Water quality data were collected at seven lakes across the United States (Figure 1). *T*_*w*_ and DO were collected using in situ sensors. Full descriptions of the sensors and protocol are included in the data product metadata provided by NEON (DP1.20264.001, NEON TSD) for *T*_*w*_ and DP1.20288.001 for DO (NEON water quality). At each lake, data were only available at one location (generally at the center, near the deepest point). For the purposes of the Challenge, unpublished data were made available to participants by NEON at a data latency of 2–3 days after collection. The *T*_*w*_ and DO NEON data products extend back to 2016, but their temporal coverage varies across sites in three ways. First, there is variability in the duration of time‐series data available for each site and variable (Appendix S1: Figure S1), ranging from 3.1 to 6.6 years (up to 1 January 2023, the beginning of our focal forecasting period). Second, at five lake sites, sensors are removed during winter due to ice formation. Finally, maintenance issues resulted in data gaps at some sites. Consequently, total data availability varied between 167 and 2154 days for each site/variable combination (Appendix S1: Figure S1).

#### Data processing and targets generation

We, as challenge organizers, converted the *T*_*w*_ and DO data supplied by NEON in near‐real time to “targets”—observations specific to the challenge—by subsetting the sensor locations, performing additional quality control, and aggregating 30‐min sensor data to daily means. We used daily mean temperature to focus the Challenge on predicting day‐to‐week dynamics in water quality, rather than subdaily dynamics. The data were subset to include only the surface measurements (top 1 m of the water column). Using only surface measurements, rather than full water column profiles, enabled intercomparison across the seven lakes, which had varying maximum depths that ranged from 3.2 to 27 m. Second, we filtered the data using the existing NEON flags (see metadata) and applied additional quality control measures (e.g., additional filtering for maximum and minimum allowable values for each variable; see Olsson, Carey, et al., 2024b). The targets data could then be used by teams to calibrate and train models and were used for forecast evaluation.

These processed target data were publicly available to all Challenge teams at a persistent URL location and were updated daily as new data became available. To further support modeling efforts by the teams, we also provided supplementary hourly water temperature profile data collected by NEON at each of the lake sites (derived from NEON DP1.20264.001, see Olsson, Carey, et al., 2024b). These supplemental data were available to teams to use in model development and training but were not used in forecast evaluation.

#### Ancillary driver data

NOAA's Global Ensemble Forecasting System (GEFS; Hamill et al., 2022) weather forecast data were made available to forecast teams via functions in the custom R package *neon4cast* (Boettiger & Thomas, 2024). NOAA weather data for all NEON sites were downloaded each day and standardized to be used as driver data and covariates in forecast models. Teams were not required to use weather covariates, but providing standardized NOAA weather forecasts ensured that the teams that used weather covariates had consistent data, and weather forecast performance was therefore not the primary driver of differences in aquatic forecast performance among model submissions. Two NOAA data products were used by forecast teams: an ensemble forecast of future weather and a historic weather product. The ensemble weather forecast consisted of 31 ensemble members up to 35 days into the future at each of the seven sites. The historic product consisted of stacked 1‐day‐ahead forecasts from each day as an estimate of observed historical conditions that was consistent with the ensemble weather forecast data available to teams to forecast (i.e., having similar biases, compared with observational weather data) and could be used to calibrate models. Teams were also able to use any other openly‐available covariate data in their forecasts, although none chose to do this.

#### Forecast submission guidelines

Challenge teams were invited to forecast *T*_*w*_ and DO in all of the lakes or in any subset of sites or variables. Forecast submissions were required to have a daily time step of the focal variable(s) over a forecast horizon of at least 1–30 days into the future and include an estimate of uncertainty in the forecast. Uncertainty could be represented by submitting a probabilistic forecast (Gneiting & Katzfuss, 2014), either in the form of a mean and a SD for a normally distributed forecast or as an ensemble forecast for which the uncertainty was represented as a series of predictions that represent a range of future conditions (Gneiting & Katzfuss, 2014). Submissions were required to follow a standardized format (Dietze et al., 2023; Thomas et al., 2023) to enable automated evaluation and processing. New forecasts were accepted every day and evaluated as new observational data became available (see Forecast evaluation).

During 2022 and 2023, we ran multiple workshops to introduce the Challenge to a cross‐section of aquatic and data scientists and managers to increase forecast submissions to this theme (Meyer et al., 2023; Olsson, Boettiger et al., 2024). In total, more than 300 people attended the workshops in person or online. Workshop materials were also available online for individuals or groups to use independently (Olsson, Boettiger et al., 2024).

#### Baseline model

Following forecast evaluation best practices (Harris et al., 2018; Lewis et al., 2022), we generated a baseline model that represents a limited (naive) understanding of the system for comparison with the submitted forecast models. It can be helpful to compare submitted forecasts with forecasts generated from baseline models as part of forecast evaluation to identify whether new methods provide additional, useful information beyond uninformed models (Jolliffe & Stephenson, 2012; Makridakis et al., 2020; Pappenberger et al., 2015). Specifically, we generated a model that assumes the forecast for a particular day‐of‐year (DOY) is equal to the mean of historical data on that DOY. The DOY baseline model assumes dynamics will follow the mean conditions for that date in previously observed years (Hyndman & Athanasopoulos, 2021; Jolliffe & Stephenson, 2012). The uncertainty in this DOY forecast was generated by calculating the SD of the past observations (see Appendix S1: Text S1). The SD of the daily average for the forecast period was used to represent the uncertainty for the whole horizon. The DOY forecast was assumed to follow a normal distribution, given by a mean and SD for each day of year calculated separately for each site and variable.

The baseline model was selected based on the observed dynamics of the variable of interest (Jolliffe & Stephenson, 2012; Pappenberger et al., 2015) as well as being a common baseline for ecological forecasts (e.g., Lewis et al., 2022; Thomas et al., 2020; Wheeler et al., 2024). The DOY model is particularly useful as a baseline when the target variable's dynamics follow a seasonal cycle (Pappenberger et al., 2015), such as variables primarily driven by meteorological forcing. A second baseline model that assumes a forecast is equal to the last observation (persistence; Jolliffe & Stephenson, 2012) was also included in submissions.

#### Forecast evaluation

Initially, forecasts were evaluated against observations using the CRPS, as implemented in the *scoringRules* R package (Jordan et al., 2019). CRPS evaluates the probability distribution of the forecast and assesses both the accuracy and precision of the forecast relative to observations and is calculated as follows:

(1) $$\text{CRPS}=\int {\left(F\left(y\right)-H\left(y-y_{\mathrm{obs}}\right)\right)}^{2}\mathrm{dy}$$

where *y* is the value for the forecasted variable, *y*_obs_ is the observation, and *F*(*y*) is the cumulative distribution function of the probabilistic forecast at the value of *y*. *H* is the indicator or step function, which is zero if *y* < *y*_obs_ and one otherwise (Jordan et al., 2019). CRPS is a generalization of mean absolute error for probabilistic forecasts and is expressed in the same units as the variable, and ranges from an optimal value of zero to infinity (Pappenberger et al., 2015). In addition, we used a *relative forecast skill* (hereafter, CRPS_skill_ or skill) metric to describe how much additional information is gained in each model over a naive baseline model. CRPS_skill_ was calculated based on the difference in CRPS score between the submitted forecast and the DOY baseline model, following Equation (2):

(2) $${\text{CRPS}}_{\text{Skill}}=\text{forecast}\_\text{score}-\mathrm{DOY}\_\text{score}$$

with positive values indicating a submitted forecast showing lower skill and higher error, relative to the DOY model, and negative values indicating that the submitted model performed better with lower error rates, as quantified using CRPS. We used this convention to ease comparison with other papers that synthesized submissions to the NEON Ecological Forecasting Challenge (e.g., Wheeler et al., 2024). We opted to focus on CRPS_skill_ relative to the DOY model rather than the persistence baseline model as the DOY model had lower average CRPS and was the better performing of the baseline models for more of the 30‐day‐ahead forecast horizons (27 out of the 30 days; Appendix S1: Figure S2).

### Analyses

We assessed the performance of the forecast models across different horizons and sites by aggregating raw CRPS_skill_ metrics at different temporal and spatial scales. To identify the best performing models per variable, we calculated the mean CRPS_skill_ aggregated across all forecast submission dates, horizons, and sites. To ensure that the comparisons among models were based on a similar number of submissions, we only included models in the analysis that had submissions for 80% of evaluated days (i.e., days with observations). We allowed teams to “catch‐up” their forecasts (i.e., submit forecasts that were not “real time” but “retroactive forecasts” following Jolliffe & Stephenson, 2012) when they missed submissions due to any issues with automated cyberinfrastructure. Retroactive forecasts could only use target data and forecasted covariates that would have been available if the forecast was generated in real time (i.e., a retroactive forecast of water temperature for 1 July 2023 only used observations before this date for model training and was driven by NOAA weather forecasts generated on 30 June 2023 or earlier). No model was represented only by retroactive forecasts. In our analysis, we removed the 16‐day‐ahead horizon from evaluation because of processing issues when downloading NOAA weather forecasts. The 16‐day‐ahead horizon had an artificially low variance in the forecast that was not present in the other horizons due to an error in the post‐processing of the weather forecast from the 6‐ to 1‐h time resolution. The 1‐ to 16‐day‐ahead forecast becomes available for download from NOAA earlier than the 17‐ to 35‐day‐ahead forecast. When combining the two sets of forecasts and temporally downscaling to a 1‐h time step, ensemble members were not matched correctly, resulting in reduced variance at the concatenation point. The processing issue was resolved during the period of evaluation, but we excluded the affected horizon, regardless, so that we could compare forecasts throughout all of 2023.

The reliability of the CIs was calculated by estimating the percentage of observations that fell within a specified CI. Reliability refers to the statistical agreement of forecast probabilities with observed relative frequencies of events (Gneiting et al., 2007; Schepen et al., 2016; see also *calibration* and *coverage*). A forecast that has perfectly reliable CIs will have the equivalent proportion of the observations falling within the CI (Jolliffe & Stephenson, 2012; Thomas et al., 2020): for example, 80% of observations falling within the 80% CI and 95% of observations falling within the 95% CI. “Underconfident” forecasts are represented by CIs that are too wide and result in more observations falling within them (e.g., 90% of observations falling within an 80% CI), whereas “overconfident” forecasts have CIs that are too narrow and fail to capture the observations (e.g., only 40% of observations falling inside an 80% CI) (following Ouellet‐Proulx et al., 2017; Thomas et al., 2020; Zwart et al., 2023). We opted to look at the 80% and 95% CIs as the 80% CI covers the bulk of the forecast distribution, and the 95% CI shows the ability of the forecast to represent the values in the tails of the distribution.

## RESULTS

### Forecast inventory

Individuals and teams submitted a total of 100,475 daily forecasts for 1‐ to 30‐day‐ahead horizons using 28 different models (Olsson, Carey, et al., 2024a) to the aquatics lake theme of the NEON Challenge in 2023. Here, we define one forecast as a collection of predictions for 1–30 days in the future for a unique combination of forecast starting date, forecast site, forecasted variable, and forecasting model. The 28 models were used in addition to the two baseline models (persistence and DOY models) submitted by Challenge organizers (*n* = 30 models total). The forecasted variables were unevenly represented in the submissions: 14 models (plus two baselines) were used to submit forecasts for both variables (*T*_*w*_, DO), 14 models were used to submit forecasts for only *T*_*w*_, and no models submitted forecasts for only DO (total model submissions for each variable: *T*_*w*_ = 30, DO = 16). Across all submissions, forecasts of water temperature for the lake sites were the most numerous (*n* = 63,189; 63% of total lake forecasts) and had a greater diversity of model classes and covariates.

The 30 *T*_*w*_ models included a range of model classes and exogenous covariates. The self‐reported model classes included empirical models (statistical and time series), ML, and PB models, as well as multimodel ensembles (MME; i.e., predictions were based on an aggregation of other model forecast submissions). Within the MMEs, forecasts were generated by combining process models, baseline and process models, empirical and baseline models, and an MME of the two baselines (Table 2; Appendix S1: Table S1). Forecast models included a range of exogenous covariates from the NOAA GEFS weather forecasts, with forecasted air temperature being the most commonly used covariate (*n* = 19; Appendix S1: Table S1). No other exogenous covariates (i.e., non‐NOAA GEFS weather covariates) were included in any model. Details of all of the models that submitted forecasts in 2023 that met the criteria for inclusion in this analysis are provided in Appendix S1: Text S1 and Olsson, Carey, et al. (2024a).

**TABLE 2:** Representation of uncertainty within the best performing water temperature (*T*_*w*_) models (sorted in descending order) that had negative mean CRPS_skill_ (i.e., outperformed the day‐of‐year baseline) over the 1‐ to 30‐day‐ahead forecast horizon.

Model	Model classification	Source of uncertainty represented				
		Driver	Parameter	Process	Initial conditions	Observation
FLARE‐GLM	PM	✕	✕	✕	✕	✕
FLARE‐GLM‐noDA	PM	✕	✕	✕	✕	✕
FLARE‐GOTM	PM	✕	✕	✕	✕	✕
XGBoost	ML	✕		✕		
Random Forest	ML	✕				
LER‐Baselines MME	MME (PB, Baseline)	✕	✕	✕	✕	✕
FLARE‐LER MME	MME (PB)	✕	✕	✕	✕	✕
FLARE‐GOTM‐noDA	PM	✕	✕	✕	✕	✕
Prophet	ML		✕	✕		
Lasso	ML	✕				

*Note*: See Table 1 for definitions of uncertainty types. Model type classification as categorized by model teams: ML, machine learning model; MME, multimodel ensemble; PM, process model. For the MME model type, the constituent model class is shown in parentheses. For the comprehensive list of uncertainty sources for all submitted models and all variables, see Appendix S1: Table S1.

**FIGURE 2:**
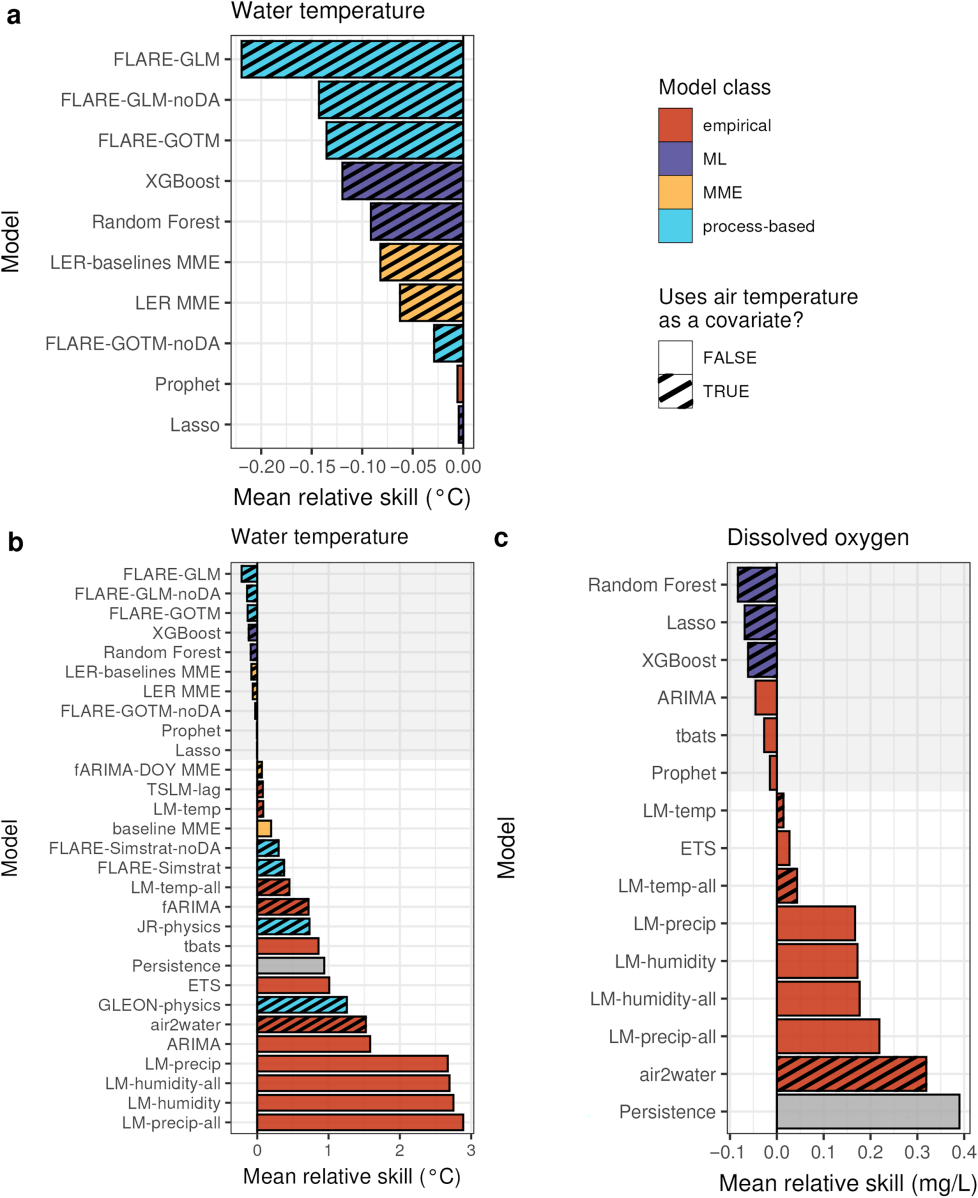
Mean relative skill (CRPS_skill_, compared with day‐of‐year [DOY] baseline model) of water temperature (*T*_*w*_) and dissolved oxygen (DO) forecasts for the submitted models (averaged across sites, submission dates, and 1‐ to 30‐day‐ahead horizons). Negative values indicate that a submitted model performed better, on average, than the DOY baseline and positive values indicate that the baseline performed better. (a) The *T*_*w*_ models that outperformed the DOY baseline as defined by CRPS_skill_; (b) all *T*_*w*_ models; (c) CRPS_skill_ for DO models. The shading of the bars indicates the model structure; color indicates model class (empirical, machine learning [ML], multimodel ensemble [MME], process), and pattern indicates the inclusion of air temperature as a covariate. A second baseline model (persistence) is shown in gray (b, c) and models that outperformed the DOY baseline are highlighted by the gray background shading. Constituent model classes of the multimodel ensemble models are given in Table 2 and Appendix S1: Table S1. CRPS, continuous rank probability score.

The 16 DO models represented less diversity in model classes and covariates than the *T*_*w*_ models (Figure 2). The model classes for the DO models included only empirical and ML models (in addition to the baseline models), and air temperature was used as a covariate in six of the 16 DO models (38%).

### How does model class affect forecast performance across all variables?

More *T*_*w*_ forecast models (*n* = 10) outperformed the DOY baseline than DO forecast models (*n* = 6; Figure 2). Only six of the submitted DO models outperformed the DOY baseline model across all forecast dates and sites (i.e., models had mean negative CRPS_skill_, with a mean between −0.01 and −0.08 mg/L aggregated across the 1‐ to 30‐day‐ahead horizon; Figure 2c). These six highest performing DO models included both ML and empirical models, of which the highest performing models were ML models that used air temperature as a covariate (Random Forest, Lasso, and XGBoost). The models that did not outperform the baseline were all empirical, and no PB models were used to forecast DO in lakes.

Unlike DO, the best performing models for water temperature (*T*_*w*_) were from the full range of model classes (Figure 2a,b). Of the 30 submitted models, 10 *T*_*w*_ forecast models outperformed the DOY baseline model when forecasts were aggregated across all sites and horizons for the year of forecasts. Across all sites and forecasts, a PB model had the best skill (Figure 2a), with a mean CRPS_skill_ of −0.22°C aggregated across the 1‐ to 30‐day‐ahead horizon. Although the overall top three models were PB models, not all PB models were high performing, as four PB models had a positive mean CRPS_skill_ (Figure 2b).

Altogether, of the different model classes used to submit forecasts of *T*_*w*_, 4 of the 8 PB models, 1 of the 13 empirical models, 2 of the 4 MME, and all 3 of the ML models outperformed the baseline DOY model on average over the year (Figure 2a). Machine learning models accounted for three models in the top 10 *T*_*w*_ forecast models, as XGBoost, Random Forest, and Lasso models all had negative CRPS_skill_. Empirical models exhibited the worst performance among the model classes, as only one (the Prophet model, Figure 2a) outperformed the DOY baseline model across all forecasts. Given the better performance of forecasts for *T*_*w*_ (10 models beating the baseline), as well as the higher diversity of model classes represented in these higher performing models (*n* = 4), further analyses for addressing Q1, Q2, and Q3 were conducted on the *T*_*w*_ forecasts only.

### Among *T*_*w*_ models, how does model class and inclusion of covariates affect performance across the forecast horizon?

Nine out of the 10 *T*_*w*_ models that outperformed the baseline model included air temperature as a covariate (Figure 2a). The specific inclusion of air temperature as a covariate appeared to confer some skill, as it was not included in any of the five lowest performing models (Figure 2b). However, the inclusion of exogenous covariates did not guarantee high performance of a model, as 10 of the models exhibiting positive CRPS_skill_ included air temperature as a covariate, as well as other NOAA weather covariates such as humidity and precipitation (Appendix S1: Text S1). There was only one model that outperformed the baseline model, the empirical Prophet model, which was based solely on observations and included no exogenous covariates (Figure 2a).

**FIGURE 3:**
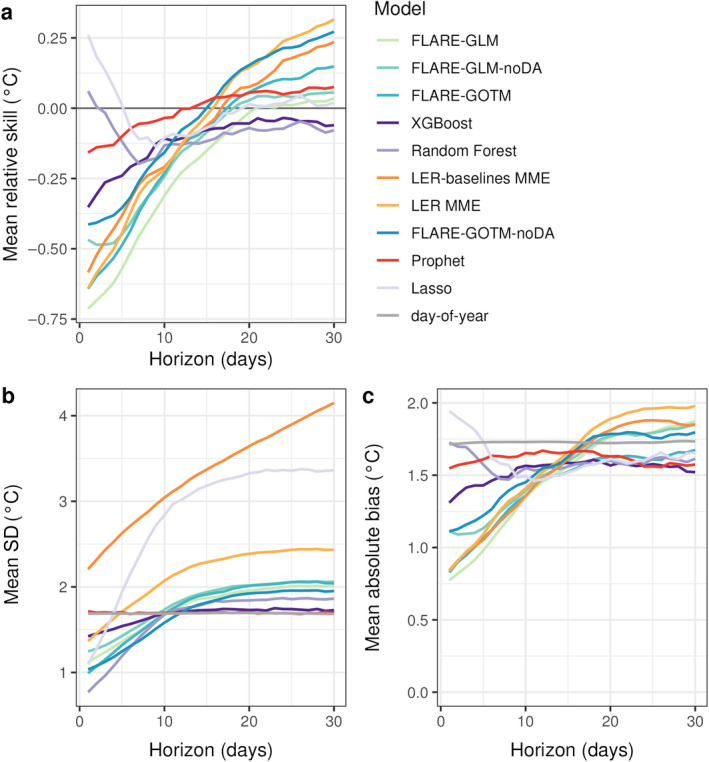
(a) Relative skill, (b) mean standard deviation (SD), and (c) mean absolute bias across the 30‐day‐ahead forecast horizon for the models that outperformed the day‐of‐year (DOY) baseline for water temperature. Relative skill was calculated as the difference in continuous rank probability score between the focal model and the DOY baseline, with negative values indicating that a submitted model performed better, on average, than the DOY baseline and positive values indicating that the baseline performed better. The metrics in each panel were averaged across all sites and forecast submission dates. Models are listed in the key in ascending order of mean skill aggregated over the forecasting period.

Focusing on *T*_*w*_, CRPS_skill_ in the most skillful forecasts generally worsened across the forecast horizon (Figure 3a), and, on average, were unable to outperform the DOY baseline at horizons of 15–25 days ahead. The exceptions to this pattern were the Lasso and Random Forest ML models, which showed improvement in skill for the first 7 or 8 days ahead and then decreases in skill at horizons longer than 8 days. Generally, the PB models and MME forecasts showed larger rates of degradation compared with the ML and empirical models (Figure 3a). The Prophet ML model exhibited the smallest degradation in skill (−0.16–0.08°C) across the 30 days, although its skill at 7‐ to 16‐day‐ahead horizons was the worst of any model that outperformed the baseline (Figure 3a). In comparison, two MME forecasts showed the largest rates of degradation (LER baselines MME and FLARE‐LER MME), from high performance at short horizons (−0.58 and −0.64°C) to poor performance at the longest horizons (0.24 and 0.32°C). Only one model had negative CRPS_skill_ across the full forecast horizon, the XGBoost ML model, which had a low rate of skill degradation across the 30 days (0.32°C; Figure 3a). The models that exhibited poorer skill throughout the 30‐day forecast horizon generally showed consistently worsening performance into the future (Appendix S1: Figure S3), although the worst performing models had poor performance irrespective of forecast horizon.

Of the 10 models that outperformed the DOY model on average, 7 models also outperformed the persistence model at all forecast horizons. Only the empirical Prophet model and the ML Lasso and Random Forest models did not outperform the persistence model at all horizons; the persistence model was better performing during the first 3 days of the forecast (Appendix S1: Figure S4). The persistence model had its highest performance at the shortest horizons (1–3 days‐ahead) and was the best performing baseline model at these horizons (Appendix S1: Figure S2).

Out of all *T*_*w*_ models that outperformed the DOY baseline (as determined by the aggregation of skill over the full forecast horizon; Figure 2a), XGBoost had negative CRPS_skill_ for the full forecast horizon, outperforming the DOY and persistence models at all horizons (Appendix S1: Figure S5). The FLARE‐GLM PB model and the Random Forest ML model had the next longest durations, where they outperformed the DOY (i.e., 19 and 27 days, respectively, over the 30‐day forecast horizon), but differed in the timing of these days. The Random Forest model had positive CRPS_skill_ at the start of the forecast horizon and FLARE‐GLM had positive relative skill at the end of the forecast period (Figure 3a), although both were only marginally worse‐performing than the baseline on the days when their CRPS_skill_ was positive. FLARE‐GLM was the most skillful model for the first 16 days of the forecast horizon, dropping only to the fourth highest performer overall at other horizons. In contrast, the best performing model at 30 days ahead, the Random Forest model, was the second worst‐performing model at 1–4 days ahead.

### To what extent is relative forecast skill affected by the inclusion of different sources of uncertainty?

Although submissions were required to include an estimate of forecast uncertainty (Thomas et al., 2023), the sources of uncertainty varied among the models. The most commonly represented source of uncertainty in *T*_*w*_ models was driver uncertainty (*n* = 22; Appendix S1: Table S1), with 13 models including only one source of uncertainty, seven models including two sources, one model including three sources, and eight models including all five sources of uncertainty (defined in Table 1).

Of the 10 *T*_*w*_ models that had mean negative skill aggregated over the forecast horizon for *T*_*w*_ (Figure 2a), seven included at least three sources of uncertainty and six included five sources (Table 2). All but one model (*n* = 9) included driver uncertainty (in the form of the NOAA GEFS weather ensembles as covariates), with parameter and process uncertainty the next most common uncertainty source included with these top models (*n* = 8 models represented this source of uncertainty). In comparison, *T*_*w*_ models that failed to outperform the baseline rarely included sources of uncertainty other than driver data uncertainty (Appendix S1: Table S1).

The degradation in relative skill for the majority of *T*_*w*_ models at longer horizons was concurrent with an increase in bias (i.e., lower accuracy; Figure 3c) and SD (i.e., lower precision; Figure 3b). The improvement in relative skill exhibited by two ML models (Lasso and Random Forest) across the first 7 days of the forecast horizon (Figure 3a) was concurrent with reductions in absolute bias (Figure 3c). Across the first 10 days, the PB models (FLARE‐GLM, FLARE‐GOTM) and MMEs that included the PB models (FLARE‐LER MME and LER baselines MME) exhibited the lowest absolute bias, which increased steadily across the horizon up to ~20 days ahead. In comparison, the forecast accuracy and to a certain extent, precision, in the Prophet, XGBoost, and Random Forest ML models degraded less, resulting in lower bias and SD at longer horizons (Figure 3c).

**FIGURE 4:**
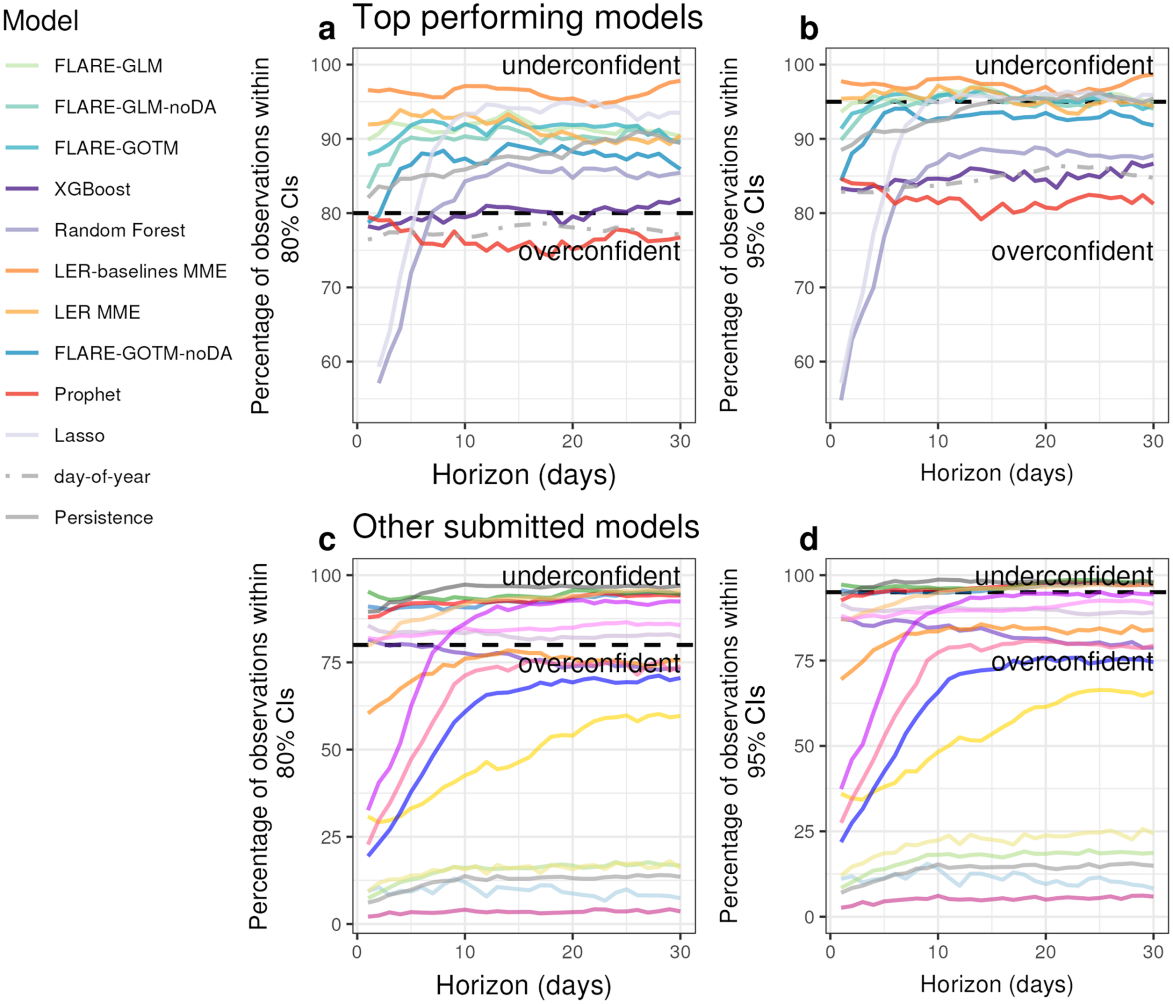
Reliability plot (percentage of observations falling within the 95% and 80% confidence intervals (CI for the water temperature models that (a, b) outperformed the day‐of‐year baseline and (c, d) those that did not (gray lines). Perfectly confident forecasts would have an equal percentage of observations within the CI as the percentage covered by the CI. Values above the dashed line indicate that the forecast is underconfident (forecast precision is too wide) and values below the line indicate that the forecast is overconfident (forecast precision is too narrow). Values above the dotted threshold indicate that the forecast is underconfident (i.e., there are too many observations falling within the specified CI) and values below the line indicate that the forecast is overconfident. Note the differences in scale between Panels (a, b) and (c, d) that show the 80% and 95% CIs, respectively.

Increased SD (i.e., greater uncertainty) across the forecast horizon may indicate a reduction in precision in the forecasts, which can degrade CRPS_skill_ and reliability of the forecast CIs. The top performing *T*_*w*_ models were primarily underconfident (Figure 4a) for the 80% CIs, meaning that >80% of observations fell within the 80% CIs. Generally, the confidence of the forecasts changed little over the horizon, especially beyond the first 5 days (Figure 4a). Beyond this horizon, only the Random Forest and Lasso ML models showed shifts in confidence beyond 5 days, becoming less overconfident and eventually becoming underconfident at horizons greater than 8 days (Figure 4). The XGBoost ML model yielded the most reliable forecasts, with 80.4% of observations in the 80% CI when averaged across horizons (Figure 4). The Prophet model was the only model that outperformed the baseline that was overconfident for the whole forecast horizon, with its uncertainty changing little across the forecast horizon (74%–79% of observations in the 80% CI; Figure 4a). The two MME models showed the highest rates of underconfidence, with 91.5% and 96.2% points falling on average into the 80% CI (Figure 4). Among the poorer performing *T*_*w*_ models, there was a greater rate of overconfidence, especially at horizons less than 7 days ahead, with 9 out of the 18 models overconfident. The rate of overconfidence increased among all models at the 95% CI (Figure 4b,d), demonstrating poor calibration for models when forecasting observations at the tails of the distribution.

### Are the patterns in performance consistent across sites?

**FIGURE 5:**
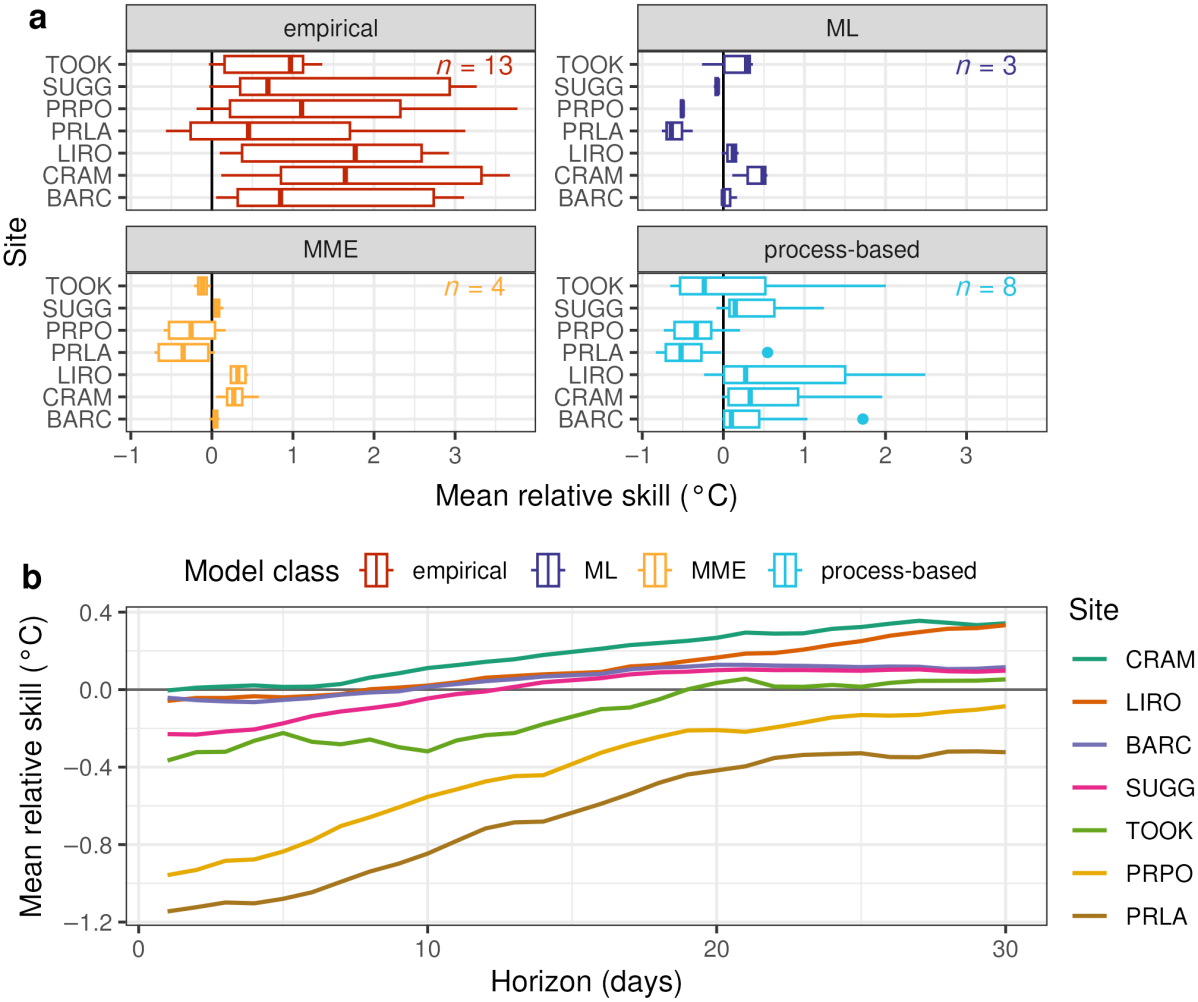
(a) Relative skill of water temperature forecasts compared with the baseline (day‐of‐year) for each site compared among model classes: Empirical, machine learning (ML), multimodel ensemble (MME), and process‐based. Negative values indicate the submitted model performed better, on average, than the baseline and positive values indicate that the baseline performed better. The *n* value indicates the number of models represented in each model class. (b) Mean relative skill for the top 10 performing models among sites across the forecast horizon.

Within model classes, *T*_*w*_ forecast CRPS_skill_ showed similar patterns among sites, with the exception of empirical models (Figure 5a). Generally, ML, PB models, and MMEs had negative CRPS_skill_ at PRLA, PRPO, and TOOK, although the latter had a limited number of forecasts given its much shorter buoy deployment duration (Appendix S1: Figure S1). In comparison, ML models, PB models, and MMEs generally exhibited positive CRPS_skill_ at SUGG, BARC, and CRAM (Figure 5a).

Mean CRPS_skill_ (from the *T*_*w*_ models that outperformed the baseline, as shown in Figure 2a) degraded across the forecast horizon for all sites, but remained negative at PRPO and PRLA for the full 30‐day horizon and at TOOK for the first 18 days (Figure 5b). In contrast, at CRAM, LIRO, BARC, and SUGG, CRPS_skill_ was negative between 1 and 12 days ahead. This better CRPS_skill_ at PRPO, PRLA, and TOOK is likely due to the relative gains against more poorly performing DOY baseline forecasts at these sites (Appendix S1: Figure S6). Focusing on the 4 months when all lakes had data availability (i.e., when all lakes had buoys deployed) versus longer time periods did not substantially alter the differences in CRPS_skill_ observed among lakes (Appendix S1: Figure S7).

**FIGURE 6:**
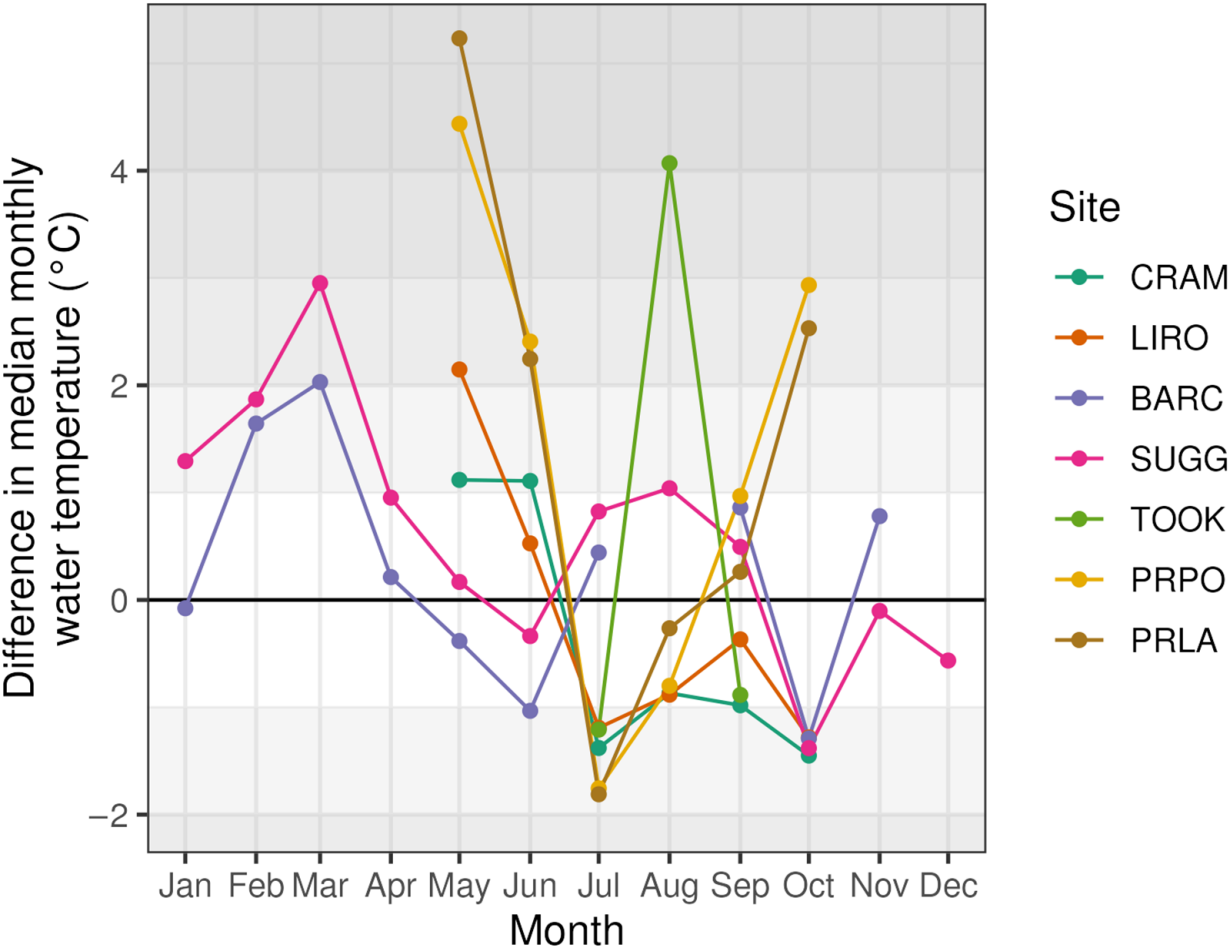
Difference in median monthly surface water temperature (depths < 1 m) between 2023 and historical observations (2015–2022) at the seven lake sites. Shaded regions show delta values that exceed 1°C from median historical conditions. Not all lakes have historical observations for the full 8‐year historical period or observations during all months.

Climate variability may have influenced why some models performed better than others in forecasting out‐of‐sample conditions. Observations for water temperatures in 2023 show that PRPO and PRLA were warmer than historical conditions represented in the DOY model, especially in May and June (Figure 6). In comparison, CRAM and LIRO, for which models performed worse than the baseline on average, exhibited water temperatures generally within around 2°C of historical conditions (Figure 6). BARC and SUGG exhibited a smaller range of water temperatures that fell within 2°C of historical conditions for all months except March (Figure 6).

## DISCUSSION

Among the 29 models that forecasted water quality variables across seven lakes, 10 models outperformed the baseline model for *T*_*w*_, and 6 for DO (Figure 2). Of the 10 best performing *T*_*w*_ models, there were 4 PB models that included multiple exogenous weather covariates, 3 ML models, 2 multimodel ensembles, and 1 empirical model, demonstrating that multiple different model classes can yield skillful forecasts for lake water temperature. Our uncertainty analysis showed that poorly performing *T*_*w*_ models were generally more overconfident, likely due to insufficient representation of uncertainty in the forecasts. Finally, model skill was inconsistent across sites for the best performing lake temperature forecast models, which may be related to site‐to‐site differences in weather. Below, we discuss how our findings addressed our research questions, with a focus on the *T*_*w*_ models.

### How do model class and model covariates affect forecast performance?

No individual model submitted to the challenge was the best performing model for both variables, although four models outperformed the baselines for both *T*_*w*_ and DO. These four models—the ML models XGBoost, Random Forest, and Lasso and the empirical model Prophet—show that a range of model types were useful for a range of variable forecasts. High‐performing models for DO were in both empirical and ML categories, although no PB or MME models were submitted for DO, necessitating further investigation of both model types to potentially improve forecast performance (Hagedorn et al., 2005; Olsson, Moore, et al., 2024). In contrast, models outperforming the baseline for *T*_*w*_ came from four model classes (ML, PB, empirical, and MMEs).

In an analysis of *T*_*w*_ models specifically (because of the higher diversity of model classes that were submitted for this variable), we found that PB models that included air temperature as a covariate performed best across all sites (Figure 2a). Air temperature is likely a key covariate for high‐performing surface water temperature forecasts because *T*_*w*_ dynamics are primarily driven by, and tightly related to, processes at the air–water interface of lakes (Piccolroaz et al., 2024; Schmid & Read, 2022). Air temperature is a causal forcing variable and is highly correlated with other key meteorological drivers (Livingstone & Padisák, 2007). PB models that used additional meteorological parameters (e.g., incoming short‐wave radiation, relative humidity, wind speed) to calculate heat fluxes to mechanistically derive water temperatures had even higher performing forecasts (Figure 2), although at some horizons the PB models were outperformed by ML models, which did not include physical processes (Figure 3a). One exception was a simple‐physics PB model that included fewer sources of uncertainty and was not able to outperform the baseline model (Appendix S1: Text S1). Altogether, our results strongly support that including the dominant drivers of water temperature (namely, air temperature) unsurprisingly improved the performance of lake water temperature forecasts.

In contrast to the *T*_*w*_ PB models, the domain‐agnostic models (i.e., models that do not include any mechanistic information about lake functioning; ML and empirical models) showed less degradation across the forecast horizon, which may be potentially due to the nondynamic nature of the methods (Appendix S1: Table S1). In comparison, the PB models were more skillful at short horizons, suggesting that forecasters might choose different *T*_*w*_ models based on the horizon needed. XGBoost, Lasso, and Random Forest ML models and the empirical Prophet model were less skillful than the PB models and PB‐MMEs in the first 10 days, but become more skillful than the PB models at horizons >10 days due to their low rates of degradation. XGBoost was the only model that outperformed the baseline across the full forecast horizon (on average for all forecasts and sites), highlighting a robust method for forecasting *T*_*w*_ at any site in our study. Our results are similar to other ecological forecasting studies: for example, domain‐agnostic models outperformed PB models in a penguin population forecasting competition in which annual populations were forecasted up to 3 years ahead (Humphries et al., 2018). Similarly, simple time‐series models have shown promise in other ecological population forecasts (Ward et al., 2014). In the NEON Challenge, the same ML and empirical models that performed well for *T*_*w*_ also performed well for DO forecasts, on average outperforming the DOY baseline, and thereby representing robust methods across multiple variables.

Reduction in the skill of *T*_*w*_ forecasts over the forecast horizon may be linked to a reduction in skill of the air temperature forecasts being used as model driver data. The Prophet model, which was the only model that outperformed the baseline that did not include air temperature as a covariate (or any covariates at all), showed less degradation in forecast performance than the overall better performing PB models, although this represents only a single model. The PB models, generally, benefit from high weather forecast skill at shorter horizons (Petchey et al., 2015; Zhou et al., 2022) but degrade in performance along with the performance of their covariates. Beyond 10 days ahead, when the weather forecasts are less skillful (Zhou et al., 2022), the PB models' performance also degraded, suggesting that ecological forecasting models requiring weather drivers may be restricted by the skill of weather forecasts. Future analyses that quantify the contribution of the weather driver accuracy and uncertainty to ecological forecast skill could determine whether this decline in skill is due to degradation in weather forecast skill or the accumulation of uncertainty from other sources.

The differences in the forecast horizons at which each *T*_*w*_ model was most skillful may present opportunities for generating MMEs or hybrid models (e.g., combining domain‐agnostic models with PB models) to exploit the strengths of multiple model types across the forecast horizon. Hybrid model approaches have shown high performance in other forecasting challenges and competitions (Clark et al., 2022; Makridakis et al., 2020), and MMEs are most successful when the individual model structures are more diverse (Dormann et al., 2018; Olsson, Moore, et al., 2024; Petropoulos et al., 2022). The performance of the MMEs in this NEON Challenge synthesis was not consistent with previous studies and other forecasting challenges, in which MMEs showed the best performance (Clark et al., 2022; Makridakis et al., 2020). For example, in forecasts of tick disease incidence, the simple model average of four individual models was better than any individual model (Clark et al., 2022), and the winner of the M4 forecasting competition (a wide‐ranging time series forecasting challenge) was a combination of statistical and empirical models (Makridakis et al., 2020). Similarly, in a recent single‐site lake study, forecasts generated by an MME composed of three PB and two baseline models outperformed the individual models across 2 years (Olsson, Moore, et al., 2024). Conversely, in this analysis, the same MME had lower relative skill, higher bias, and higher uncertainty than some of the individual models from which it was derived (Figure 2). This discrepancy in MME performance could be caused by poor calibration in the individual models at some of the lake sites. The individual models included in this study were almost all underconfident (Figure 4), which resulted in very large uncertainty in the MMEs and likely contributed to their poor performance, as MME forecasts have been shown to be most successful when the individual constituent models are slightly overconfident (Hagedorn et al., 2005; Wang et al., 2023). Methods such as trimming, where distributions are narrowed, could help constrain MME uncertainty, increasing the overall skill of these forecasts (Howerton et al., 2023).

Finally, the differences among forecasting models, especially within model classes, can be interpreted in the context of model relatedness. For example, six of the seven PB models were 1‐D hydrodynamic models that share meteorological drivers (Olsson, Moore, et al., 2024). These six PB models included three unique 1‐D hydrodynamic models (GLM, GOTM, and Simstrat, see Appendix S1) with and without an ensemble Kalman filter data assimilation method. As a result of the PB models using similar equations for modeling some components of lake ecosystems (e.g., well‐established surface energy balance equations) and the shared data assimilation approaches, the PB models are not entirely independent representations. Similarly, many of the empirical models were based on the same structures with differing drivers. Here, we focused on analyzing the results for broad classes of models (PB, empirical, and ML) rather than within‐model classes to reduce the impact of any of model relatedness on the analysis. Future work can build on lessons learned in the climate modeling community to interpret multimodel analyses in the context of quantifying model independence and similarity (Pathak et al., 2023; Pennell & Reichler, 2011).

### To what extent is relative forecast skill affected by the inclusion of different sources of uncertainty?

Our synthesis suggests that representation of forecast uncertainty is important for determining the overall forecast performance of probabilistic *T*_*w*_ forecasts. The top performing *T*_*w*_ models often included multiple sources of uncertainty (up to *n* = 5; Table 2), unlike the lower performing models, which frequently only included driver uncertainty. Consequently, many poor performing models were overconfident in their predictions, suggesting there was insufficient uncertainty included in those forecasts. By omitting parameter and process uncertainty, the forecasts fail to acknowledge the inability of the models to completely capture the ecological and stochastic processes being modeled and that even complex process models are an approximation of reality (Dietze, 2017). These results suggest that driver uncertainty alone is not a sufficient representation of the total uncertainty, especially given that weather forecasts are themselves often overconfident at shortest forecast horizons (1–7 days; Zhou et al., 2022). When these weather forecasts are used as driver data for overfitted lake models (Zwart et al., 2023), overconfidence in water quality forecasts is even more likely to occur. Overconfidence of forecasts was also reported in a forest phenology forecast synthesis, in which forecasts that included covariates were overconfident at shorter horizons (Wheeler et al., 2024). In our analysis, the Lasso and Random Forest ML models, which only included driver uncertainty, showed performance improvements from 1 to 8 days ahead (Figure 3), as the uncertainty from the weather forecasts increased and the water temperature forecasts became less overconfident (Figure 4). Furthermore, the ML XGBoost model, which included process uncertainty in addition to driver uncertainty, outperformed the other ML models at shorter horizons. Improving the representation of uncertainty for many of the models that failed to outperform the baseline could be achieved by additionally quantifying: (1) the uncertainty from the chosen model through the inclusion of parameter and/or process uncertainty; or (2) from the measurements through the inclusion of initial conditions or observational uncertainty (see Table 1).

Improving the representation of uncertainty in forecasts, as quantified by the reliability of forecast CIs, is important for management (Crochemore et al., 2021; Ramos et al., 2013). The use of ecological forecasts by decision makers is likely to improve if forecast uncertainty is well quantified and CIs are appropriate (Buizza, 2008; Nadav‐Greenberg & Joslyn, 2009; Ramos et al., 2013). Underconfidence and overconfidence limit the use of forecasts for management, as underconfident forecasts provide too wide of a range of potential future conditions and overconfident forecasts underestimate the possible range of conditions, with both leading to inappropriate management actions (Crochemore et al., 2021). Consequently, our results suggest that including more than one source of uncertainty may help increase the usability of forecasts as decision support tools.

### Is model forecast performance consistent across sites?

*T*_*w*_ forecast performance varied among sites, with the relative gain in skill likely due to the lower performance of baseline models at some lakes, especially at PRPO and PRLA, two lakes in North Dakota. The DOY baseline model had the lowest performance at PRPO and PRLA, potentially because 2023 conditions in these two lakes were substantially different from historical observations, resulting in a lower performing baseline forecast (Figure 6; Appendix S1: Figure S6). This is consistent with a previous single‐model forecasting study (FLARE‐GLM) that also showed improved performance above a DOY baseline for these two sites, especially at shorter horizons (Thomas et al., 2023). Differences from historical conditions that exceeded 3°C resulted in poor DOY baseline performance in that study. Our results suggest that if there is a divergence of water temperature of this magnitude, using a PB or ML model provides a much stronger forecasting approach than a baseline model. All model classes except the empirical model class showed better performance compared with the DOY baseline at PRLA and PRPO as well as at TOOK, to a lesser extent. As environmental conditions further exceed historical means due to global change, models that only consider patterns from long‐term historical observations may be less valuable than models that are able to infer ecological processes or use recently‐observed data in generating forecasts.

### Value and refinements for forecasting challenges

Forecasting challenges provide a compelling opportunity to learn about ecological predictability over gradients of time, space, ecological level of organization, and forecasting methods. The submissions from 30 models (including two baselines) to the aquatics lake theme of the NEON Challenge covered a range of model classes and approaches. However, since the NEON Challenge was open to the community and we did not specifically guide the types of submissions, the breadth of models was not exhaustive and therefore some questions remain. Specifically, quantifying the value of different covariates to different models (e.g., XGBoost, linear models, Random Forest) would be best done by comparing forecasts with the same modeling approach but with differing covariates and quantitatively seeing how forecast skill changes with their addition or removal. It is possible that this “model selection” was done by teams before forecasts were submitted and that the final model submitted to the Challenge was the optimal structure, but we cannot know from the submitted metadata whether these models represent each team's “best” attempt at producing a forecast.

We also saw uneven representation in the variables being forecasted, with more submitted forecasts of *T*_*w*_ than DO. We identified several potential factors that contributed to this uneven representation. First, NEON Challenge training materials were focused on lake temperature forecasting, which may have skewed submissions to this variable because participants in workshops may have been more likely to modify pre‐existing code for submitting a new model type to *T*_*w*_, rather than develop new code for DO submissions. Second, water temperature may have been an easier, more “introductory” forecast target variable as there are well‐established mechanistic processes linked to driver datasets (e.g., meteorology) that were made readily available for teams to use. Conversely, the drivers of DO concentrations are much more complex, drawing from physical, chemical, and biological processes (Carey, 2023; Hanson et al., 2006; Langman et al., 2010) that vary by timescale (Hanson et al., 2006; Langman et al., 2010) and are likely to be more or less important depending on lake mixing (Robbins et al., 2024), trophic status (Steinsberger et al., 2020), and lake size (Langman et al., 2010). To use these additional driver data to forecast model lake DO processes, forecasts of those drivers must first be generated before they can be used in a model submitted to the Challenge.

Overall, our conclusions about the best performing model are limited to mean surface water temperature (the target variable chosen by the Challenge organizers), as forecasts at other depths or temporal aggregations may lead to different conclusions. For example, in contrast to our findings about surface temperature, Thomas et al. (2020) found persistence forecasts of bottom water temperature performed better than a process model because of the low variability in temperature below the thermocline. Our analyses motivate future work that focuses on different depths and temporal aggregations, as motivated by the needs of forecast users. When using forecasting methods for environmental management, the appropriateness of the forecast target (e.g., surface water temperature vs. chance of lake mixing), in addition to the chosen models should be evaluated to ensure that models, and forecast output are fit for their management purpose (Bokulich & Parker 2021; Parker, 2020). Applying methods and approaches from one application in a new situation, without accessing the fitness‐for‐purpose, could result in misplaced confidence or harmful outcomes (Parker, 2020).

Nonetheless, the forecasting approaches shown in this synthesis could provide a valuable starting point for developing forecasts for management decision‐making or as inputs into other models and decision‐support tools (e.g., Carey et al., 2022), for example, using a water temperature forecast as an input into an algal bloom risk model. For the NEON lake sites specifically, although not actively managed, water temperature forecasts of these lakes may help to optimize NEON sampling protocols, for example, by forecasting the lake ice‐on dates and therefore maximizing the deployment of the water quality buoys that have to be removed during winter ice cover or to anticipate a water quality impairment event for higher frequency spatial sampling.

The NEON Challenge also sets the stage for future forecasting model analyses. For example, future work could address whether the inclusion of exogenous covariates in models produces forecasts that are overconfident at shorter horizons for other ecological variables, which could be corrected using multiple sources of uncertainty. Similarly, it would be useful to investigate whether the domain‐agnostic models that outperformed the baseline for DO and *T*_*w*_ perform similarly well when forecasting other ecological variables. The spatial and temporal extent of NEON data, as well as the range of ecological variables on which data are collected, provides a suite of opportunities to continue to investigate these questions and as a platform to grow the field of ecological forecasting.

## CONCLUSION

Our synthesis of more than 100,000 submissions to the NEON Forecasting Challenge demonstrates that several model classes were able to outperform a DOY baseline model to forecast water temperature and dissolved oxygen across seven lake sites, providing insight into optimal forecasting approaches for different contexts. Water temperature models that included air temperature as an exogenous covariate and those that included multiple sources of uncertainty generally performed well and came from PB, empirical, ML, and multimodel ensemble model classes. The relative skill of these models was shown to be highest at sites that exhibited conditions outside of historical observations. These forecasting methods are likely to become increasingly valuable for guiding decision‐making in a world in which ecosystems are become more variable and continue to move outside of historically observed conditions. Overall, our results highlight the value of forecasting challenges to advance the development of ecological forecasts for both theory and management.

## AUTHOR CONTRIBUTIONS

R. Quinn Thomas, Freya Olsson, and Cayelan C. Carey designed and developed the NEON EFI Aquatics Challenge. R. Quinn Thomas, Carl Boettiger, and Freya Olsson developed cyberinfrastructure. All coauthors contributed to forecasts. Freya Olsson, Cayelan C. Carey, and R. Quinn Thomas developed the synthesis and analysis approach with feedback from all coauthors. Freya Olsson led the manuscript writing, supported by Cayelan C. Carey and R. Quinn Thomas. All coauthors contributed to the text describing the forecasting approaches, provided feedback, and approved the final manuscript.

## CONFLICT OF INTEREST STATEMENT

The authors declare no conflicts of interest.

## Supporting information


Appendix S1.

## Data Availability

Model code used to generate forecasts (Olsson, Carey, et al., 2024a), data analyzed in the synthesis (Olsson, Carey, et al., 2024b), and the code used to generate figures and results in this manuscript (Olsson, Thomas, et al., 2024) are available on Zenodo: https://doi.org/10.5281/zenodo.13750779, https://doi.org/10.5281/zenodo.11087208, https://doi.org/10.5281/zenodo.11093206, respectively.
