# Supplementary material for: What can we learn from 100,000 freshwater forecasts? A synthesis from the NEON Ecological Forecasting Challenge
Source: Ecol Appl. 2025 Feb 12;35(1):e70004. doi: 10.1002/eap.70004 (PMC11816007; doi:10.1002/eap.70004)
Supplement: Supplementary file 1 — Appendix S1. [file EAP-35-e70004-s001.pdf]

Appendix S1 for *"What can we learn from 100,000 freshwater forecasts? A synthesis from the NEON Ecological Forecasting Challenge" Ecological Applications*

Freya Olsson, Cayelan C. Carey, Carl Boettiger, Gregory Harrison, Robert Ladwig, Marcus F. Lapeyrolerie, Abigail S. L. Lewis, Mary E. Lofton, Felipe Montealegre-Mora, Joseph S. Rabaey, Caleb J. Robbins, Xiao Yang, R. Quinn Thomas

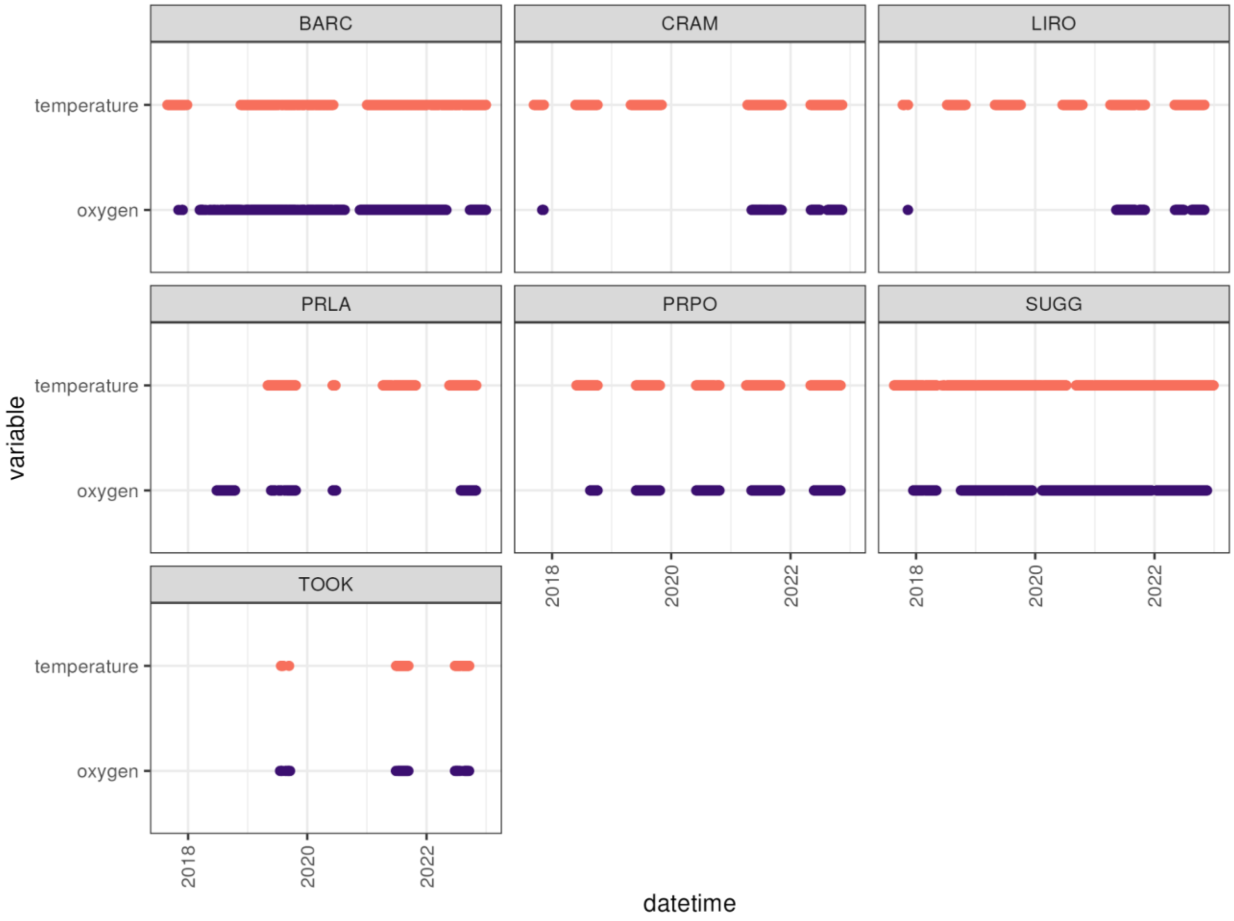

Figure S1. Time series of the availability of water temperature and dissolved oxygen targets data for each lake site and variable within the NEON Forecasting Challenge. Each panel shows data from a site denoted by the four-character NEON site code.

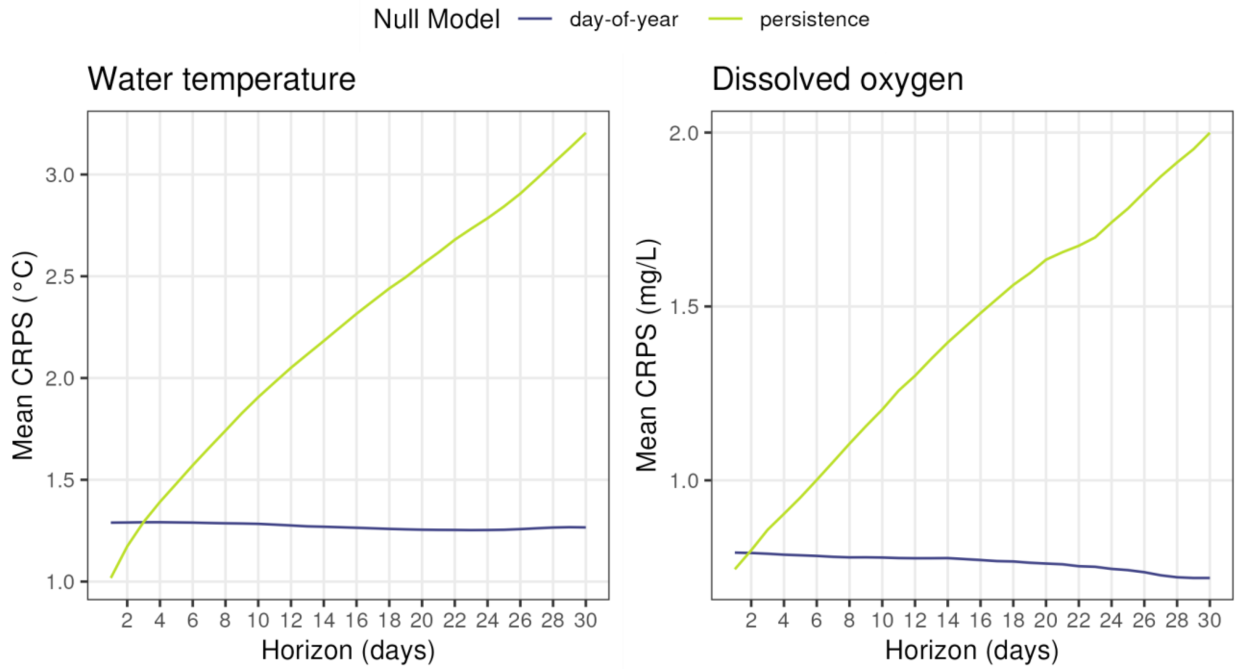

Figure S2. Mean Continuous Rank Probability Score (CRPS) of baseline models for water temperature and dissolved oxygen (averaged for all forecasts and sites) across the 1-30 day-ahead forecast horizon.

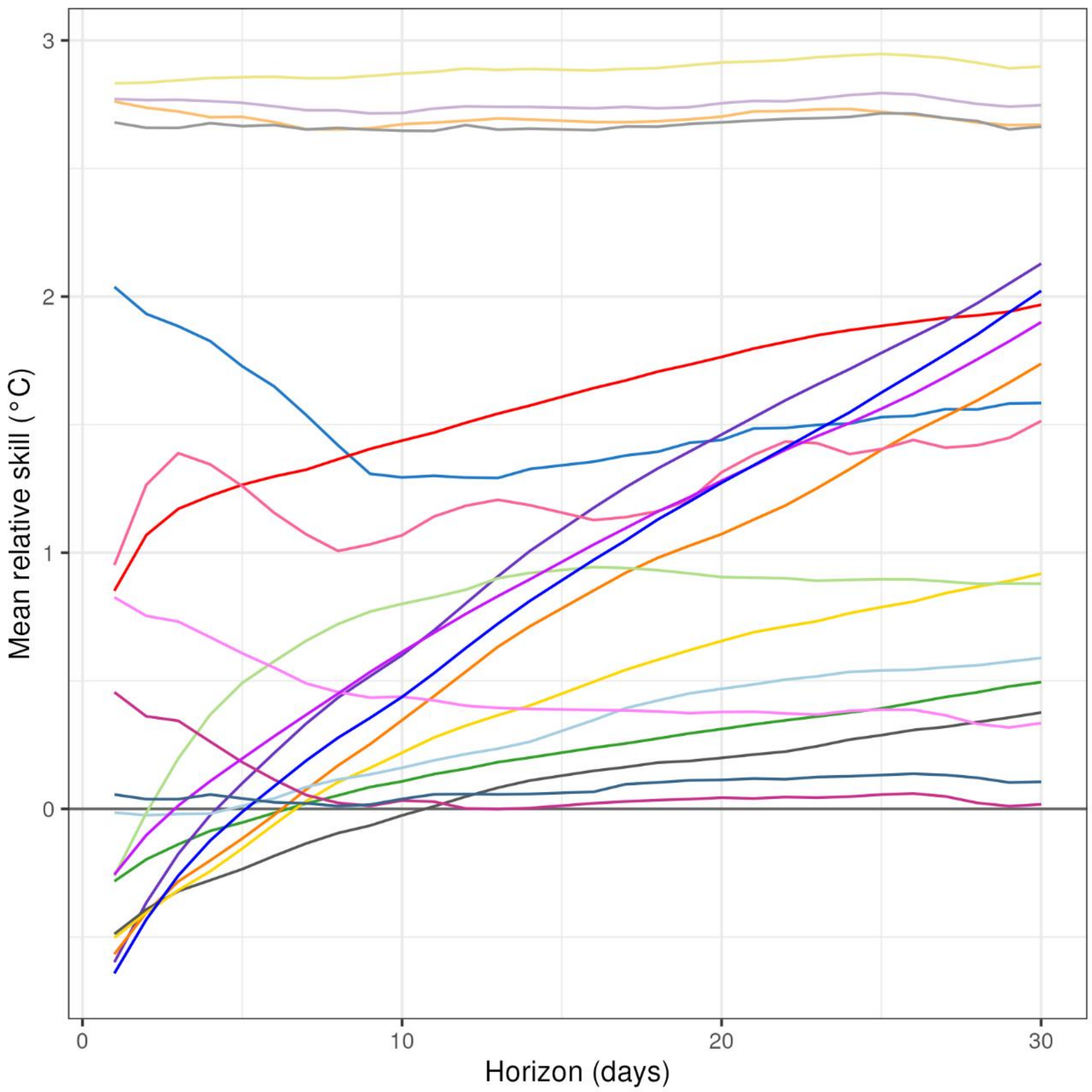

Figure S3. Mean relative skill of temperature (Tw) models that did not out-perform the day-of-year baseline (on average for all forecasts and sites) across the 30 day-ahead forecast horizon. Relative skill was calculated as the difference in continuous rank probability score (CRPS) between the focal model and the persistence baseline. The skill was averaged across all sites and forecast submission dates. Positive relative skill indicates that the baseline performed better (submitted model had larger forecast error) and negative relative skill indicates the submitted model performed better.

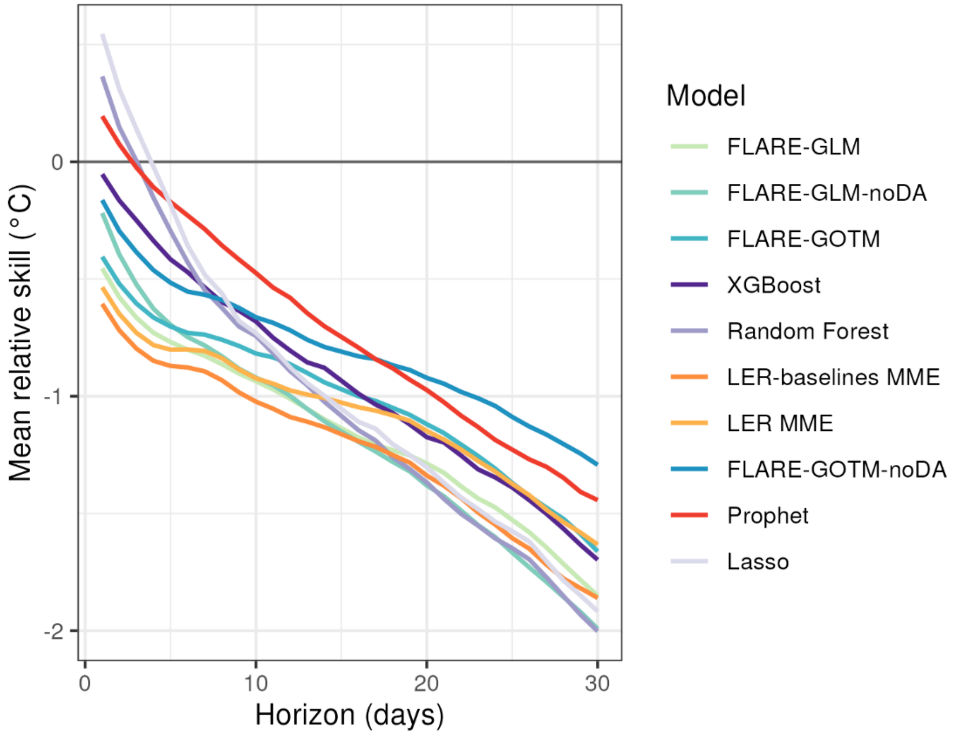

Figure S4. Mean forecast skill relative to the persistence model across the 30 day-ahead forecast horizon for the models that outperformed the day-of-year (DOY) baseline for water temperature. Relative skill was calculated as the difference in continuous rank probability score (CRPS) between the focal model and the persistence baseline. The skill was averaged across all sites and forecast submission dates. Positive relative skill indicates that the baseline performed better (submitted model had larger forecast error) and negative relative skill indicates the submitted model performed better. Models are listed in the legend in ascending order of mean skill relative to DOY aggregated over the forecasting period.

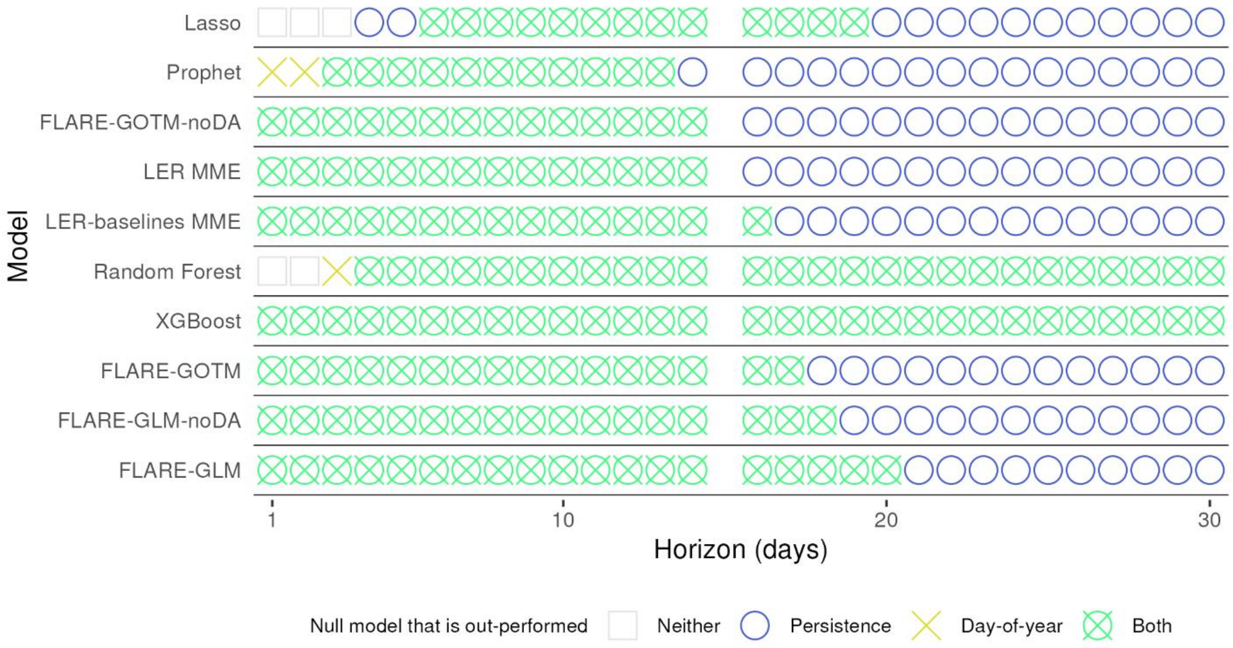

Figure S5. Comparison of the models that outperformed the day-of-year baseline model on average at each forecast horizon with both baseline models. The shape and colour of the points describes which baseline model, if any, was outperformed by the submitted model.

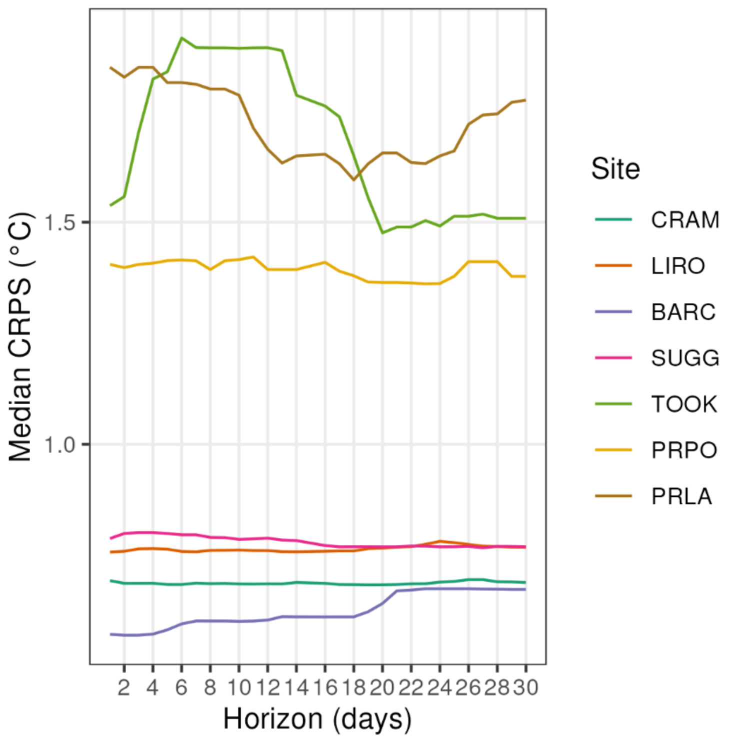

Figure S6. Median water temperature forecast performance (continuous rank probability score, CRPS, °C) of the baseline day-of-year across the forecast 1-30 day horizon at the seven lake sites.

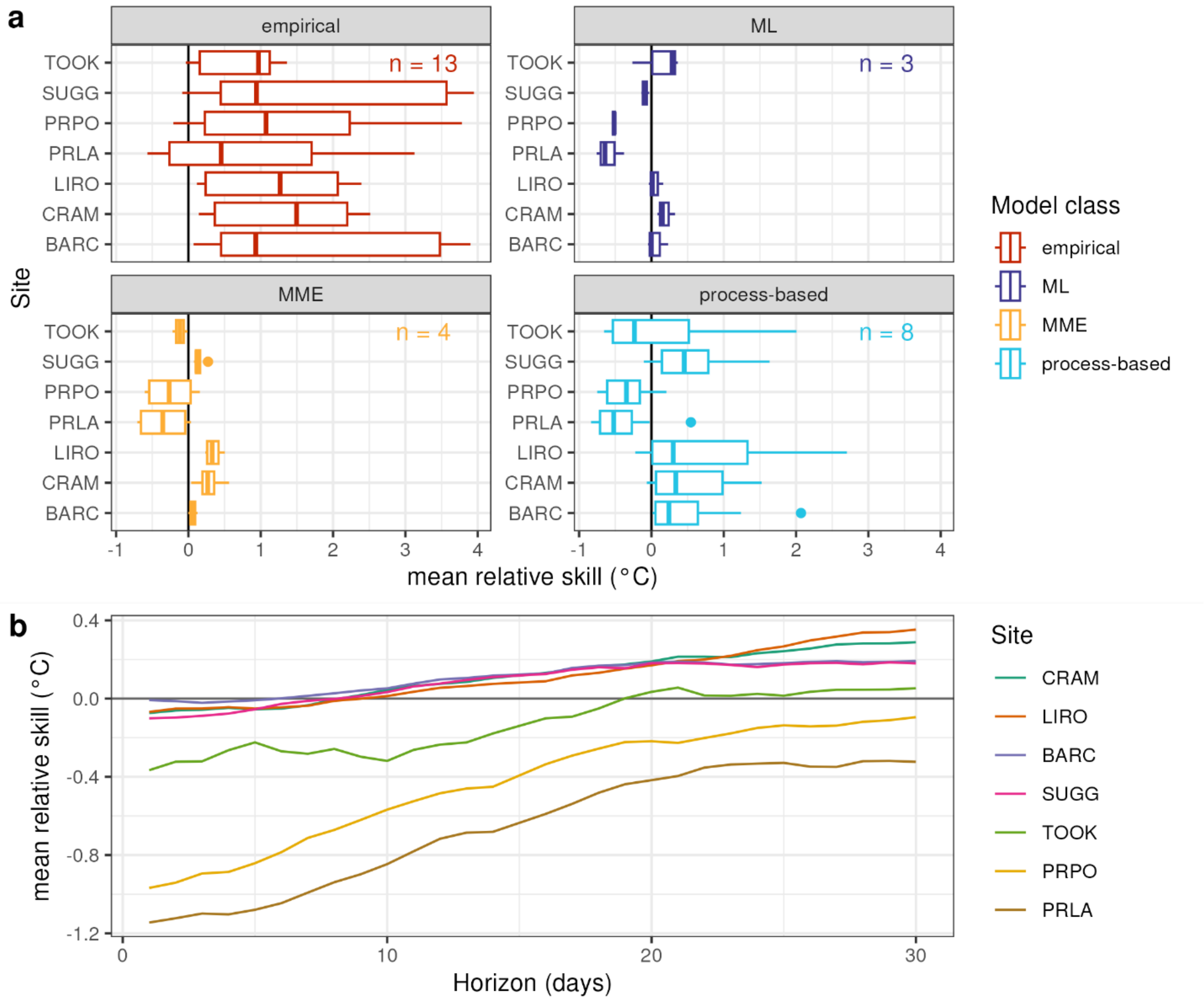

Figure S7. a) Relative skill of water temperature forecasts compared to the baseline (day-of-year) for each site compared among model classes (empirical, machine-learning (ML), multi-model ensembles (MME), and process). Relative skill was calculated as the difference in continuous rank probability score (CRPS) between the focal model and the persistence baseline. The skill was averaged across all sites and forecast submission dates. Positive relative skill indicates that the baseline performed better (submitted model had larger forecast error) and negative relative skill indicates the submitted model performed better. The *n* value indicates the number of models represented in each model class. b) Average relative skill for the top ten performing models among sites across the forecast horizon. Duration of forecasts was consistent among sites.

Table S1. A summary of model structure (type, covariates), sources of uncertainty, and use of historical data to produce initial conditions and update parameters for 30 forecast models submitted to the aquatics theme of the NEON Forecasting Challenge. A full description of the models can be found in Supplementary Text 1. Definitions for uncertainty types can be found in Table 2 in the main manuscript. *MME = multi-model ensemble, constituent model names and classes are shown in parentheses).

Model Name	Model Type	Forecast Variables	Model Covariates	Includes Initial Conditions?	Is Model Dynamic?	Sources Of Uncertainty Represented	Updates Parameters?
**Air2Water**	Empirical	Temperature, Oxygen	Air Temperature	No	No	Driver	Yes
**Baseline MME* (Day-of-year; Persistence)**	MME (baseline)	Temperature	-	Yes	Yes	Process	No
**Prophet**	Empirical	Temperature, Oxygen	-	No	Yes	Parameter, Process	Yes
**Day-Of-Year**	Baseline	Temperature, Oxygen	-	No	No		No
**fARIMA**	Empirical	Temperature	Air Temperature	Yes	Yes	Process, Driver	Yes
**fARIMA-DOY MME* (fARIMA, Day-of-year)**	MME (Empirical)	Temperature	Air Temperature	Yes	Yes	Process	Yes
**LER MME* (FLARE-GLM; FLARE-GOTM; FLARE-Simstrat)**	MME (Process)	Temperature	Air Temperature, Air Pressure, Relative Humidity, Surface Downwelling Longwave, Surface Downwelling Shortwave, Precipitation, Eastward Wind, Northward Wind	Yes	Yes	Parameter, Process, Initial Conditions, Driver, Observation	Yes

**LER-Baselines MME* (FLARE-GLM; FLARE-GOTM; FLARE-Simstrat; Day-of-year; Persistence)**	MME (Process; Baseline)	Temperature	Air Temperature, Air Pressure, Relative Humidity, Surface Downwelling Longwave, Surface Downwelling Shortwave, Precipitation, Eastward Wind, Northward Wind	Yes	Yes	Parameter, Process, Initial Conditions, Driver, Observation	Yes
**FLARE-GLM**	Process	Temperature	Air Temperature, Air Pressure, Relative Humidity, Surface Downwelling Longwave, Surface Downwelling Shortwave, Precipitation, Eastward Wind, Northward Wind	Yes	Yes	Parameter, Process, Initial Conditions, Driver, Observation	Yes
**FLARE-GLM- noDA**	Process	Temperature	Air Temperature, Air Pressure, Relative Humidity, Surface Downwelling Longwave, Surface Downwelling Shortwave, Precipitation, Eastward Wind, Northward Wind	No	No	Parameter, Process, Initial Conditions, Driver, Observation	No
**FLARE-GOTM**	Process	Temperature	Air Temperature, Air Pressure, Relative Humidity, Surface Downwelling Longwave, Surface Downwelling Shortwave, Precipitation, Eastward Wind, Northward Wind	Yes	Yes	Parameter, Process, Initial Conditions, Driver, Observation	Yes

**FLARE-GOTM-noDA**	Process	Temperature	Air Temperature, Air Pressure, Relative Humidity, Surface Downwelling Longwave, Surface Downwelling Shortwave, Precipitation, Eastward Wind, Northward Wind	No	No	Parameter, Process, Initial Conditions, Driver, Observation	No
**FLARE-Simstrat**	Process	Temperature	Air Temperature, Air Pressure, Relative Humidity, Surface Downwelling Longwave, Surface Downwelling Shortwave, Precipitation, Eastward Wind, Northward Wind	Yes	Yes	Parameter, Process, Initial Conditions, Driver, Observation	Yes
**FLARE-Simstrat-noDA**	Process	Temperature	Air Temperature, Air Pressure, Relative Humidity, Surface Downwelling Longwave, Surface Downwelling Shortwave, Precipitation, Eastward Wind, Northward Wind	No	No	Parameter, Process, Initial Conditions, Driver, Observation	No
**TSLM-Lag**	Empirical	Temperature	Air Temperature	No	No	Process, Driver	Yes
**JR-Physics**	Process	Temperature	Air Temperature	Yes	Yes	Driver	No
**GLEON-Physics**	Process	Temperature	Air Temperature, Relative Humidity, Surface Downwelling Shortwave, Eastward Wind, Northward Wind	Yes	Yes	Process	No

**Persistence**	Baseline	Temperature, Oxygen	-	Yes	Yes	Process	No
**ARIMA**	Empirical	Temperature, Oxygen	-	Yes	Yes	Process	Yes
**ETS**	Empirical	Temperature, Oxygen	-	Yes	Yes	Process	Yes
**LM-Humidity**	Empirical	Temperature, Oxygen	Relative Humidity	No	No	Driver	Yes
**LM-Humidity-All**	Empirical	Temperature, Oxygen	Relative Humidity	No	No	Driver	Yes
**Lasso**	Machine-learning	Temperature, Oxygen	Air Temperature, Air Pressure, Relative Humidity, Surface Downwelling Longwave, Surface Downwelling Shortwave, Precipitation Flux, Northward Wind, Eastward Wind	No	No	Driver	No
**LM-Precip**	Empirical	Temperature, Oxygen	Precipitation	No	No	Driver	Yes
**LM-Precip-All**	Empirical	Temperature, Oxygen	Precipitation	No	No	Driver	Yes
**Random Forest**	Machine-learning	Temperature, Oxygen	Air Temperature, Air Pressure, Relative Humidity, Surface Downwelling Longwave, Surface Downwelling Shortwave, Precipitation, Eastward Wind, Northward Wind	No	No	Driver	No
**TBATS**	Empirical	Temperature, Oxygen	-	Yes	Yes	Process	Yes

**LM-Temp**	Empirical	Temperature, Oxygen	Air Temperature	No	No	Driver	Yes
**LM-Temp-All**	Empirical	Temperature, Oxygen	Air Temperature	No	No	Driver	Yes
**XGBoost**	Machine-learning	Temperature, Oxygen	Air Temperature, Surface Downwelling Shortwave, Relative Humidity	No	No	Process, Driver	Yes

Text S1. This supplementary text contains descriptions of the submitted models included in this paper. The model descriptions are provided by the forecast teams and include a description of the model's structure and general forecasting methodology. The directory within the archived model code is provided (see the Zenodo archive in Olsson *et al.*, 2024).

air2water (air2waterSat_2)

The air2water model is a linear model fit using the function lm() in R and uses air temperature as a covariate. The model fits water temperature (Tw) as a function of air temperature (Ta) and generates a forecast using forecasted water temperatures, following:

$$Tw = Ta * \beta_0 + \beta_1$$

where β_0 is a slope term and β_1 is an intercept. The uncertainty in drivers was obtained by using the 31 ensemble members from the NOAA GEFS forecast.

From these forecasted water temperatures, the dissolved oxygen concentration was estimated assuming 100% saturation of oxygen within the water (based on the temperature and elevation-dependent state calculation). To estimate the concentration of dissolved oxygen at saturation, the Eq.Ox.conc() in the *rMR* R package was used.

This model was used to forecast water temperature and dissolved oxygen concentration at the seven lake sites, with the model fitted separately for each site.

Team members: This model was generated as an example model by EFI-NEON Challenge Organisers.

Archive directory: neon4cast-example

Baseline MME (Baseline_ensemble)

The Baseline MME is a multi-model ensemble (MME) comprised of the two baseline models (day-of-year, persistence) submitted by Challenge organisers. To generate the MME, an ensemble forecast was generated by sampling from the submitted models (either from the ensemble members in the

case of the persistence, or from the distribution for the day-of-year forecasts). The forecast included 200 ensemble members, represented equally across the 2 individual models (100 per forecast). The steps to generate the MME were:

1. Access submitted forecasts for the site and variable of interest (lake temperatures only) from the submissions S3 bucket.

2. Subset by individual model ID.

3. If the forecast is a distributional forecast: sample from the distribution using the forecasted mean and standard deviation to generate a sample of n = 100.

4. If the forecast is an ensemble forecast: subsample the existing individual forecast ensemble members to generate n = 100 ensemble members. The parameter numbers (ensemble members) were consistent across the forecast horizon.

Only sites with all individual forecasts present were submitted (a site, variable, and date had to be represented by both models).

This model was used to forecast water temperature in the lake sites (BARC, CRAM, LIRO, PRLA, PRPO, SUGG, TOOK). See information about the individual forecast models for information of forecast uncertainty representation in each of the forecasts.

Team members: Freya Olsson

Archive directory: NEON-simple-baselines-FO/Models/baselines_ensemble.R

Prophet model (cb_prophet)

The Prophet model is an empirical model, specifically a non-linear regression model that includes seasonality effects (Taylor & Letham, 2018). The model relies on Bayesian estimation with an additive white noise error term:

y(t) = g(t) + s(t) + h(t) + \epsilon_t,

where g is a piecewise linear 'growth' term (with changepoints estimated by the algorithm), s is a seasonal effect (Fourier term), h is the effect of 'holidays', and epsilon is the white noise (error term). The

model does not include any covariate. We use the implementation of the Prophet model provided in the *darts* Python package (Herzen et al., 2022). See https://github.com/unit8co/darts

This model was used to forecast water temperature and dissolved oxygen concentration in the seven lake sites, with the model fitted separately for each site.

Archive directory: forecasts-darts-framework

Team members: Carl Boettiger, Marcus Francois Lapeyrolerie, Felipe Montealegre-Mora

Day-of-year (climatology)

The day-of-year (climatology) is a baseline model that assumes that forecasted conditions will be the same as the average of historical observations for that day-of-year (DOY). For each variable/site combination, the model calculates the mean (μ) and the standard deviation (σ) of the historical observations for each DOY. We assume that σ is consistent across the forecast horizon and so the median DOY σ is calculated for each new forecast (which can change between forecast dates but not across a forecast horizon).

Because of differences in sensor deployment (e.g., some lake sensors are removed in winter), not all DOYs have observations. Missing DOY means are filled using a linear interpolation, as long as at least two DOYs have values during the forecast period.

For the year 2023, the forecasts for each DOY do not change among forecast dates as no new data were collected during the forecast period (1 January 2023 - 31 December 2023) that would contribute to updated means or standard deviations.

This model was used to forecast water temperature and dissolved oxygen concentration in the seven lake sites, with the model fitted separately for each site.

Team members: this model was generated as a baseline model by EFI-NEON Challenge Organisers

Archive directory: neon4cast-baselines-main/models/aquatics_climatology.R

fARIMA (fARIMA)

The fARIMA is an empirical model that fits an ARIMA model using the *fable* R package (O'Hara-Wild, Hyndman, & Wang 2023) as a function of a linear model with air temperature. The default ARIMA() function automatically chooses the best ARIMA model for the time-series, using a step-wise procedure.

The process uncertainty is generated from the standard deviation in the residuals of the fitted model. We could not assume a normal distribution in residuals and so opted to generate an ensemble forecast using a bootstrap approach within the generate() function from *fable*. In addition, we used the 31 ensemble members from the NOAA GEFS as driver uncertainty. For each NOAA ensemble member, an ensemble forecast with six ensemble members was generated using the generate() function resulting in a total of 31 x 6 = 186 ensemble members per forecast.

Not all sites have observations for all days due to differences in maintenance (e.g., some lake sites have sensors removed in winter). Therefore, to account for the difference in the time since last observation, the forecast was started at the day after the last observation, and the horizon modified to cover up to 30 days into the future from the forecast date. During this 'catch-up' period, the pseudo-observation of air temperature used in model training was used to generate water temperature rather than forecasted air temperature.

This model was used to forecast water temperature in the seven lake sites, with the model fitted separately for each site.

Team members: Freya Olsson, R. Quinn Thomas

Archive directory: NEON-simple-baselines-FO/Models/ARIMA_model.R

fARIMA-DOY MME (fARIMA_clim_ensemble)

The fAMIRA-DOY MME is a multi-model ensemble (MME) composed of two empirical models: an ARIMA model (fARIMA) and day-of-year model. To generate the MME, an ensemble forecast was generated by sampling from the submitted models' ensemble members. The forecast

included 200 ensemble members, represented equally across the two individual models (n=100). The steps to generate the MME were:

1. Access submitted forecasts for the site and variable of interest (lake temperatures only) from the submissions S3 bucket.

2. Subset by individual model ID.

3. Subsample the existing individual forecast ensemble members to generate 100 ensemble members. The parameter numbers (ensemble members) were consistent across the forecast horizon.

4. Only sites with all individual forecasts present were submitted (a site, variable and date had to be represented by both models).

This model was used to forecast water temperature in the seven lake sites, with the model fitted separately for each site. See information about the individual forecast models for information of forecast uncertainty representation in each of the forecasts.

Team members: Freya Olsson

Archive directory: NEON-simple-baselines-FO/Models/fARIMA_clim_ensemble.R

LER MME (flare_ler)

The LER MME is a multi-model ensemble (MME) derived from the three process models from FLARE (FLARE-GLM, FLARE-GOTM, and FLARE-Simstrat). To generate the MME, an ensemble forecast was generated by sampling from the submitted models' ensemble members. The forecast included 198 ensemble members, represented equally across the 3 individual models (n=66). The steps to generate the MME were:

1. Access submitted forecasts for the site and variable of interest (lake temperatures only) from the submissions S3 bucket.

2. Subset by individual model ID.

3. Subsample the existing individual forecast ensemble members to generate 66 ensemble members. The parameter numbers (ensemble members) were consistent across the forecast horizon.

4. Only sites with all individual forecasts present were submitted (a site, variable and date had to be represented by all 3 models).

This model was used to forecast water temperature in six lake sites (BARC, CRAM, LIRO, PRLA, PRPO, SUGG), but not TOOK, with the model fitted separately for each site. See information about the individual forecast models for information of forecast uncertainty representation in each of the forecasts.

Team members: Freya Olsson

Archive directory: NEON-simple-baselines-FO/Models/flare_ler_ensemble.R

LER-baselines MME (flare_ler_baselines)

The LER-baselines model is a multi-model ensemble (MME) comprised of the three process models from FLARE (FLARE-GLM, FLARE-GOTM, and FLARE-Simstrat) and the two baseline models (day-of-year, persistence), submitted by Challenge organisers. To generate the MME, an ensemble forecast was generated by sampling from the submitted model's ensemble members (either from an ensemble forecast in the case of the FLARE models and persistence, or from the distribution for the day-of-year forecasts). The forecast included 200 ensemble members, represented equally across the 5 individual models (40 per forecast). The steps to generate the MME were:

1. Access submitted forecasts for the site and variable of interest (lake temperatures only) from the submissions S3 bucket.

2. Subset by individual model ID.

3. If the forecast is a distributional forecast: sample from the distribution using the forecasted mean and standard deviation to generate a sample of n = 40.

4. If the forecast is an ensemble forecast: subsample the existing individual forecast ensemble members to generate n = 40 ensemble members. The parameter numbers (ensemble members) were consistent across the forecast horizon.

5. Only sites with all individual forecast present were submitted (a site, variable, and date had to be represented by all 5 models).

This model was used to forecast water temperature in six of the lake sites (BARC, CRAM, LIRO, PRLA, PRPO, SUGG), but not TOOK. See information about the individual forecast models for information of forecast uncertainty representation in each of the forecasts.

Team members: Freya Olsson

Archive directory: NEON-simple-baselines-FO/Models/ler_baselines_ensemble.R

FLARE-GLM (flareGLM)

The FLARE-GLM is a forecasting framework that integrates the General Lake Model hydrodynamic process model (GLM; Hipsey et al., 2019) and data assimilation algorithm to generate ensemble forecasts of lake water temperature. FLARE's ensemble-based forecasting algorithm generates forecasts using GLM that quantifies the uncertainty from driver data (weather forecasts from NOAA's Global Ensemble Forecasting System; Hamill et al., 2022), initial conditions, model process, and model parameters and then samples from these sources of uncertainty to generate probability distributions for water temperature at multiple lake or reservoir depths (see Thomas et al., 2020).

Daily forecasts were generated for the lake sites using the following steps: Step 1) access the FLARE-GLM forecasts from the day before (or, in the case of the first forecast, following a 60 day spin-up); Step 2) use this prediction to initialise a GLM run that starts 5 days ago and runs to current day; Step 3) use the ensemble Kalman filter (Evensen, 2003) to assimilate new observations collected over the past 5 days to update GLM's states and parameters; and Step 4) use the updated states and parameters as initial conditions for a 1- to 30 day-ahead forecast that starts today. Each forecast includes 256 ensemble members that quantify the uncertainty from driver data (weather forecasts), initial conditions, model process, and model parameters.

Driver uncertainty: GLM requires the following weather covariates obtained from NOAA GEFS: air temperature, air pressure, relative humidity, wind speed (calculated from the north and east wind

speeds), precipitation, and incoming shortwave and incoming longwave radiation. The water balance method was set to include no inflows or outflows and maintain a water level. Bathymetry data for the lakes were obtained from NEON. Uncertainty from drivers was generated based on the 31 ensemble members from NOAA GEFS.

Initial conditions uncertainty: Initial conditions uncertainty was based on the spread of model states on Day 0 of the forecast that was set by spread in the 256 ensemble members following data assimilation on Day 0.

Model process uncertainty: Process uncertainty was generated by adding random noise to each ensemble, drawing from a normal distribution with a standard deviation of 0.75 °C (after Thomas et al., 2020).

Model parameter: parameter uncertainty was generated using a unique parameter value assigned to each of the 256 ensemble members that was determined through data assimilation. The parameters that are tuned in the data assimilation algorithm are specific to the hydrodynamic model. In total, two parameters were tuned in the data assimilation process: lw_factor (longwave radiation scaling factor), and sed_temp_mean (annual mean sediment temperature, °C).

Forecasts of daily surface water temperature were generated from the profiles output from FLARE-GLM by averaging temperatures forecasted in the top 1 m of the water column as a “surface” forecast. FLARE-GLM outputs forecasts for 00:00:00 and this is given as the daily forecast to the Challenge.

This model was used to forecast water temperature at the seven lake sites with the model parameters calibrated separately for each site. Additional information about FLARE configuration can be found in Thomas et al. (2020) and Thomas et al. (2023).

Team members: Freya Olsson, R. Quinn Thomas, Cayelan C. Carey

Archive directory: NEON-forecast-code/workflows/default

FLARE-GLM-noDA (flareGLM_noDA)

FLARE-GLM-noDA uses the same configuration as FLARE-GLM with the exception of the data assimilation (DA) algorithm. Within the noDA configuration, model states and parameters were not updated prior to forecast generation. Model parameters were calibrated before using observations of water temperatures and then brought ‘online’ to generate real-time forecasts using forecast drivers (NOAA weather data). Parameter uncertainty was calculated (as in FLARE-GLM), but the distributions were not updated between forecasts. The parameters calibrated were lw_factor (longwave radiation scaling factor), and sed_temp_mean (annual mean sediment temperature, °C).

Forecasts of daily surface water temperature were generated from the profiles output from FLARE-GLM-noDA by averaging temperatures forecasted in the top 1 m of the water column as a “surface” forecast. FLARE-GLM-noDA outputs forecasts for 00:00:00 and this is given as the daily forecast to the Challenge.

This model was used to forecast water temperature at the seven lake sites, with the model parameters calibrated separately for each site. Additional information about FLARE configuration can be found in Thomas et al., (2020) and Thomas et al. (2023).

Team members: Freya Olsson, R. Quinn Thomas

Archive directory: NEON-forecast-code/workflows/default

FLARE-GOTM (flareGOTM)

FLARE-GOTM uses the same principles and overarching framework as FLARE-GLM, with the hydrodynamic model replaced with the General Ocean Turbulence Model (GOTM). GOTM is a 1-D hydrodynamic turbulence model (Umlauf et al., 2005) that estimates water column temperatures. The integration of FLARE and GOTM was achieved using the LakeEnsemblR R package (Moore et al., 2021). Sources of uncertainty remain the same and are generated using equivalent methods. The parameters that were tuned in the data assimilation algorithm were specific to the hydrodynamic model and in the case of GOTM are swr_scale (short-wave radiation scaling factor) and/or wind_scale (wind

speed, u10, scaling factor), depending on the site's sensitivity. See FLARE-GLM for a full description of the sources of uncertainty and the forecast generation method.

Forecasts of daily surface water temperature were generated from the profiles output from FLARE-GOTM by averaging temperatures forecasted in the top 1 m of the water column as a "surface" forecast. FLARE-GLM outputs forecasts for 00:00:00 and this is given as the daily forecast to the Challenge.

This model was used to forecast water temperature in 6 of the lake sites (BARC, CRAM, LIRO, PRLA, PRPO, SUGG), but not TOOK, with the model parameters calibrated separately for each site. Additional information about FLARE configuration can be found in Thomas et al., (2020) and Thomas et al. (2023).

Team members: Freya Olsson, R. Quinn Thomas

Archive directory: NEON-forecast-code/workflows/ler

FLARE-GOTM-noDA (flareGOTM_noDA)

FLARE-GOTM-noDA uses the same configuration as FLARE-GLM with the exception of the data assimilation (DA) algorithm. Within the noDA configuration, model states and parameters are not updated prior to forecast generation. Model parameters were calibrated before using observations of water temperatures and then brought 'online' to generate real-time forecasts using forecast drivers (NOAA weather data). Parameter uncertainty was calculated (as in FLARE-GOTM) but the distributions were not updated between forecasts. The parameters calibrated were swr_scale (short-wave radiation scaling factor), and/or wind_scale (wind speed, u10, scaling factor).

Forecasts of daily surface water temperature were generated from the profiles output from FLARE-GOTM-noDA by averaging temperatures forecasted in the top 1 m of the water column as a "surface" forecast. FLARE-GOTM-noDA outputs forecasts for 00:00:00, which is submitted as the daily forecast to the Challenge.

This model was used to forecast water temperature in six of the lake sites (BARC, CRAM, LIRO, PRLA, PRPO, SUGG), but not TOOK, with the model parameters calibrated separately for each site.

Additional information about FLARE configuration can be found in Thomas et al. (2020) and Thomas et al. (2023).

Team members: Freya Olsson, R. Quinn Thomas

Archive directory: NEON-forecast-code/workflows/ler

FLARE-Simstrat (flareSimstrat)

FLARE-Simstrat uses the same principles and overarching framework as FLARE-GLM with the hydrodynamic model replaced with Simstrat. Simstrat is a 1-D hydrodynamic turbulence model (Goudsmit et al., 2002) that estimates water column temperatures. The integration of FLARE and Simstrat was achieved using the LakeEnsemblR R package (Moore et al., 2021). Sources of uncertainty remain the same and are generated using equivalent methods. The parameters that are tuned in the data assimilation algorithm are specific to the hydrodynamic model and in the case of Simstrat were p_sw_water (short-wave radiation scaling factor) and/or f_wind (wind speed scaling factor), depending on the site's sensitivity. See FLARE-GLM for a full description of the sources of uncertainty and the forecast generation method.

Forecasts of daily surface water temperature were generated from the profiles output from FLARE-Simstrat by averaging temperatures forecasted in the top 1 m of the water column as a "surface" forecast. FLARE-Simstrat outputs forecasts for 00:00:00, which is submitted as the daily forecast for the Challenge.

This model was used to forecast water temperature at the seven lakes, with the model parameters calibrated separately for each site. Additional information about FLARE configuration can be found in Thomas et al. (2020) and Thomas et al. (2023).

Team members: Freya Olsson, R. Quinn Thomas

Archive directory: NEON-forecast-code/workflows/ler

FLARE-Simstrat-noDA (flareSimstrat_noDA)

FLARE-Simstrat-noDA uses the same configuration as FLARE-Simstrat with the exception of the data assimilation (DA) algorithm. Within the noDA configuration, model states and parameters were not updated prior to forecast generation. Model parameters were calibrated before using observations of water temperatures and then brought 'online' to generate real-time forecasts using forecast drivers (NOAA weather data). Parameter uncertainty was calculated (as in FLARE-Simstrat) but the distributions were not updated between forecasts. The parameters calibrated were p_sw_water (incoming short-wave radiation scaling factor), and/or f_wind (wind speed scaling factor).

Forecasts of daily surface water temperature were generated from the profiles output from FLARE-Simstrat-noDA by averaging temperatures forecasted in the top 1 m of the water column as a "surface" forecast. FLARE-Simstrat-noDA outputs forecasts for 00:00:00 and this is given as the daily forecast to the Challenge.

This model was used to forecast water temperature at the seven lake sites, with the model parameters calibrated separately for each site. Additional information about FLARE configuration can be found in Thomas et al. (2020) and Thomas et al. (2023).

Team members: Freya Olsson, R. Quinn Thomas

Archive directory: NEON-forecast-code/workflows/ler

TSLM-lag (fTSLM_lag)

This is a simple time series linear model in which water temperature is a function of air temperature of that day and the previous day's air temperature. The TSLM was fit using the TSLM() function from the *fable* R package (O'Hara-Wild M, Hyndman R, Wang E, 2023).

The process uncertainty is generated from the standard deviation in the residuals of the fitted model. We could not assume a normal distribution in residuals and so opted to generate an ensemble forecast using a bootstrap approach within the generate() function from *fable*. In addition, we used the 31 ensemble members from the NOAA GEFS as driver uncertainty. For each NOAA ensemble member, an

ensemble forecast with six ensemble members was generated using the generate() function resulting in a total of 31 x 6 = 186 ensemble members per forecast.

Not all sites have observations for all days due to differences in maintenance (e.g., some lake sites have sensors removed in winter). Therefore, to account for the difference in the time since last observation, the forecast was started at the day after the last observation, and the horizon modified to cover up to 30 days into the future from the forecast date. During this 'catch-up' period the pseudo-observation of air temperature, used in model training, was to generate water temperature rather than forecasted air temperature.

This model was used to forecast water temperature at the seven lake sites, with the model fitted separately for each site.

Team members: Freya Olsson, R. Quinn Thomas

Archive directory: NEON-simple-baselines-FO/Models/TSLM_lags.R

JR-physics (GLEON_JRabaey_temp_physics)

The JR-physics model is a simple process model based on the assumption that surface water temperature should trend towards equilibration with air temperature with a lag factor.

Initial conditions for the model were set using the most recently available temperature data for each site. Forecasted water temperature was calculated as:

$$Tw_{t+1} = Tw_t + l(Ta_{t+1} - Tw_t)$$

where *Tw* is surface water temperature, *Ta* is forecasted air temperature, and *l* is the air-water equilibration lag factor. *l* was set to 0.2 for all sites.

Each forecast is generated using the 31 ensemble air temperature forecasts from the NOAA GEFS weather forecast. The model is iteratively fit each day as new data are generated by NEON.

Driver uncertainty was included by using the 31 ensemble members from the NOAA GEFS weather forecast. No uncertainties from initial conditions or model process were included in the overall forecast uncertainty.

This model was used to forecast water temperature at the seven lake sites, with the model fitted separately for each site.

Team members: Joseph Rabaey

Archive directory: Neon4cast-JR-Physics

GLEON-physics (GLEON_physics)

A simple, process-based model was developed to replicate the water temperature dynamics of a surface water layer *sensu* Chapra (2008). The model focus was only on quantifying the impacts of atmosphere-water heat flux exchanges on the idealized near-surface water temperature dynamics:

$$\frac{\partial T}{\partial t} = \frac{1}{\Delta z}\frac{(Q + H)}{c_p \rho_w}$$

where T is water temperature, t is time (fixed time step of 3600 s), Δz is the thickness of the near-surface layer which is assumed to be 1 m, Q is the net heat flux, H is internal heat generation due to incoming short-wave radiation, c_p is the heat capacity of water, and ρ_w is water density. Q represents the amount of energy from short-wave radiation that is absorbed directly in the surface layer:

$$Q = (1 - \alpha)Q_{sw}$$

with α as a constant albedo of 0.1. The net heat flux H is the sum of four terms:

$$H = H_{lw} + H_{lwr} + H_v + H_c,$$

where the terms on the right-hand side represent incoming long-wave radiation, emitted long-wave radiation from the water, the latent heat flux, and the sensible heat flux, respectively.

The heat fluxes were derived using the formulations from Livingstone and Imboden (1989), Goudsmit et al. (2002), and Verburg and Antenucci (2010). Note that the latent and sensible heat fluxes were calculated by including the actual surface area of the respective lake. To replicate the heat flux dynamics, the model used mean forecasted air temperature, relative humidity, air pressure, short-wave radiation, and wind speed from NOAA GEFS. Air vapor pressure was quantified from air temperature and relative humidity. Cloud cover was calculated using the empirical formulation from Martin and

McCutcheon (1998). Whenever water temperatures became less than the freezing point temperature of water (assumed to be 0 °C), water temperatures were set to 0 °C.

We approximated the water temperature of the next time step using an explicit Euler forward scheme, and also by including an error term on the right-hand side to account for stochastic fluctuations:

$$T_{t+1} = T_t + \frac{\Delta t}{\Delta z}\frac{(Q+H)}{c_p \rho_w} + N(\mu, \sigma)$$

where μ was set to 0 °C, and σ to 0.05 °C. For every prediction, we ran 100 model runs to quantify process uncertainty through the error term. No uncertainties from initial conditions, drivers, or parameter estimations were included in the overall forecast uncertainty.

This model was used to forecast water temperature at the seven lake sites, with the model fitted separately for each site.

Team members: Robert Ladwig, Xiao Yang

Archive directory: NEON-simple-baselines-main_RL

Persistence (persistenceRW)

The persistence (persistenceRW; random walk) is a baseline model that assumes, on average, conditions over the forecast horizon will be the same as the last observation, with uncertainty driven by a random walk process.

$$y_{T+1} = y_T + e_{T+1}$$

where y_T is today's observation or forecast, e_{T+1} is random noise, and y_{T+1} is the next day's forecast. The uncertainty (e_{T+1}) in the persistence model forecasts was generated using a bootstrapping method with no assumption placed on the distribution of the forecast. We assumed that future uncertainty will be drawn from the same distribution of the residual error in the fit to historical data. We fit the model to historical observations, using the RW() (Random walk) function in the *fable* R package (version 0.3.2; O'Hara-Wild et al., 2022), and the model error or residual (e) was calculated between the model and observations. At each timestep, a value of e_{T+1} was drawn from the distribution of these historic error values for each

ensemble member. Overall, 200 ensemble members were generated for each timestep using this method using the *fable* generate() function and a bootstrap value of 200 (number of ensemble members).

This model was used to forecast water temperature and dissolved oxygen concentration at the seven lake sites, with the model fitted separately for each site and variable.

Team members: this model was generated as a baseline model by EFI-NEON Challenge Organisers.

Archive directory: neon4cast-baselines-main/models/aquatics_persistenceRW.R

ARIMA (tg_arima)

The tg_arima model is an AutoRegressive Integrated Moving Average (ARIMA) model fit using the function auto.arima() from the *forecast* package in R (Hyndman et al. 2023; Hyndman et al., 2008). This is an empirical time series model with no covariates. The model is fit every day as new data are made available, and is fit separately for each site/variable combination. For sites/variables where all observations were non-negative, we set the Box-Cox transformation parameter (lambda) in forecast::auto.arima() to “auto”, allowing a Box-Cox transformation to be automatically selected. Forecasts were generated based on the model fit using the forecast::forecast() function, and were submitted as normal distributions using the mean and standard deviation of the forecast output.

This model was used to forecast water temperature and dissolved oxygen concentration at the seven lake sites, with the model fitted separately for each site.

Team members: Abigail S.L. Lewis, Caleb J. Robbins

Archive directory: Forecast_submissions/Generate_forecasts/ARIMA

ETS (tg_ets)

The tg_ets model is an Error, Trend, Seasonal (ETS) model fit using the function ets() from the *forecast* package in R (Hyndman et al. 2023; Hyndman et al., 2008). This is an empirical time series model with no covariates. The model is fit every day as new data are made available, and is fit separately

for each site/variable combination. We interpolated all missing data in the time series for each site/variable combination using forecast::na.interp(). For sites/variables where all observations are non-negative, we set the Box-Cox transformation parameter (lambda) in *forecast::na.interp()* to “auto”, allowing a Box-Cox transformation to be automatically selected. Forecasts were generated based on the model fit using the forecast::forecast() function, and were submitted as normal distributions using the mean and standard deviation of the forecast output.

This model was used to forecast water temperature and dissolved oxygen concentration at the seven lake sites, with the model fitted separately for each site.

Team members: Abigail S.L. Lewis, Caleb J. Robbins

Archive directory: Forecast_submissions/Generate_forecasts/ETS

TBATS (tg_tbats)

The tg_tbats model is a TBATS (Trigonometric seasonality, Box-Cox transformation, ARMA errors, Trend and Seasonal components) model fit using the function tbats() from the *forecast* package in R (Hyndman et al. 2023; Hyndman et al., 2008). This is an empirical time series model with no covariates. The model is fit every day as new data are made available, and is fit separately for each site/variable combination. We interpolated all missing data in the time series for each site/variable combination using forecast::na.interp(). For sites/variables where all observations are non-negative, we set the Box-Cox transformation parameter (lambda) in *forecast::na.interp()* to “auto”, allowing a Box-Cox transformation to be automatically selected. Forecasts were generated based on the model fit using the forecast::forecast() function, and were submitted as normal distributions using the mean and standard deviation of the forecast output.

This model was used to forecast water temperature and dissolved oxygen concentration at the seven lake sites, with the model fitted separately for each site.

Team members: Abigail S.L. Lewis, Caleb J. Robbins

Archive directory: Forecast_submissions/Generate_forecasts/TBATS

LM-humidity (tg_humidity_lm)

The tg_humidity_lm model is a linear model fit using the function lm() in R. This is a very simple model with only one covariate: relative humidity. The model is fit every day as new data are made available, and is fit separately for each site/variable combination.

Driver uncertainty was included by using the 31 ensemble members from the NOAA GEFS weather forecast. No uncertainties from initial conditions or model process were included in the overall forecast uncertainty.

This model was used to forecast water temperature and dissolved oxygen concentration at the seven lake sites, with the model fitted separately for each site.

Team members: Abigail S.L. Lewis, Caleb J. Robbins

Archive directory: Forecast_submissions/Generate_forecasts/humidity_lm

LM-humidity-all (tg_humidity_lm_all_sites)

The tg_humidity_lm_all_sites model is a linear model fit using the function lm() in R. This is a very simple model with only one covariate: relative humidity. The model is fit every day as new data are made available, and is fit across all sites, using site ID as a factor in the regression.

Driver uncertainty was included by using the 31 ensemble members from the NOAA GEFS weather forecast. No uncertainties from initial conditions or model process were included in the overall forecast uncertainty.

This model was used to forecast water temperature and dissolved oxygen concentration at the seven lake sites, with the model fitted for all sites together.

Team members: Abigail S.L. Lewis, Caleb J. Robbins

Archive directory: Forecast_submissions/Generate_forecasts/humidity_lm_all_sites

LM-precip (tg_precip_lm)

The tg_precip_lm model is a linear model fit using the function lm() in R. This is a very simple model with only total precipitation used as a model covariate. The model is fit every day as new data are made available, and is fit separately for each site/variable combination.

Driver uncertainty was included by using the 31 ensemble members from the NOAA GEFS weather forecast. No uncertainties from initial conditions or model process were included in the overall forecast uncertainty.

This model was used to forecast water temperature and dissolved oxygen concentration at the seven lake sites, with the model fitted separately for each site.

Team members: Abigail S.L. Lewis, Caleb J. Robbins

Archive directory: LM-precip: Forecast_submissions/Generate_forecasts/precip_lm

Code repository:

https://github.com/eco4cast/Forecast_submissions/blob/main/Generate_forecasts

LM-precip-all (tg_precip_lm_all_sites)

The tg_precip_lm_all_sites model is a linear model fit using the function lm() in R. This is a very simple model with only one covariate: total precipitation. The model is fit every day as new data are made available, and is fit across all sites, using site ID as a factor in the regression.

Driver uncertainty was included by using the 31 ensemble members from the NOAA GEFS weather forecast. No uncertainties from initial conditions or model process were included in the overall forecast uncertainty. This model was used to forecast water temperature and dissolved oxygen concentration at the seven lake sites, with the model fitted for all sites together.

Team members: Abigail S.L. Lewis, Caleb J. Robbins

Archive directory: LM-precip: Forecast_submissions/Generate_forecasts/precip_lm_all_sites

LM-temp (tg_temp_lm)

The tg_temp_lm model is a linear model fit using the function lm() in R. This is a very simple model with only one covariate: air temperature. The model is fit every day as new data are made available, and is fit separately for each site/variable combination.

Driver uncertainty was included by using the 31 ensemble members from the NOAA GEFS weather forecast. No uncertainties from initial conditions or model process were included in the overall forecast uncertainty.

This model was used to forecast water temperature and dissolved oxygen concentration in the seven lake sites, with the model fitted separately for each site.

Team members: Abigail S.L. Lewis, Caleb J Robbins

Archive directory: Forecast_submissions/Generate_forecasts/temp_lm

LM-temp-all (tg_temp_lm_all_sites)

The tg_temp_lm_all_sites model is a linear model fit using the function lm() in R. This is a very simple model with only one covariate: air temperature. The model is fit every day as new data are made available, and is fit across all sites, using site ID as a factor in the regression.

Driver uncertainty was included by using the 31 ensemble members from the NOAA GEFS weather forecast. No uncertainties from initial conditions or model process were included in the overall forecast uncertainty.

This model was used to forecast water temperature and dissolved oxygen concentration at the seven lake sites, with the model fitted for all sites together.

Team members: Abigail S.L. Lewis, Caleb J. Robbins

Archive directory: Forecast_submissions/Generate_forecasts/temp_lm_all_sites

Random Forest (tg_randfor)

Random Forest is a machine learning model that is fitted with the ranger() function in the *ranger* R package (Wright & Ziegler 2017) within the *tidymodels* framework (Kuhn & Wickham 2020). The

model drivers are unlagged air temperature, air pressure, relative humidity, surface downwelling longwave and shortwave radiation, precipitation, and northward and eastward wind. Only data prior to 2023-01-01 were used for any model training; similarly, model fits were not updated with any 2023 data when generating forecasts in 2023. Hyperparameters were selected for each site using 10-fold cross validation (repeated 5 times per site), selecting the hyperparameter combination with the lowest average RMSE. The number of trees was set to 500 but we tuned two hyperparameters for a) the minimum node size for each tree and b) the number of randomly selected predictors. Model predictions are independent in time. The random forest model predicts observations for every NOAA GEFS ensemble member and forecast horizon of the predicted drivers, so only driver uncertainty is represented.

This model was used to forecast water temperature and dissolved oxygen concentration in the seven lake sites, with the model fitted separately for each site.

Team members: Abigail S.L. Lewis, Caleb J. Robbins

Archive directory: Forecast_submissions/Generate_forecasts/tg_randfor

Lasso (tg_lasso)

Lasso is a machine learning model implemented in the same workflow as tg_randfor, but with different hyperparameter tuning. The model drivers are unlagged air temperature, air pressure, relative humidity, surface downwelling longwave and shortwave radiation, precipitation, and northward and eastward wind. Only data prior to 2023-01-01 were used for any model training; similarly, model fits were not updated with any 2023 data when generating forecasts in 2023. Hyperparameters were selected for each site using 10-fold cross validation (repeated 5 times per site), selecting the hyperparameter combination with the lowest average RMSE. Lasso regressions were fitted with the function glmnet() in the package *glmnet* (Tay et al. 2023), where the regularization hyperparameter (lambda) is tuned and selected with 10-fold cross validation.

This model was used to forecast water temperature and dissolved oxygen concentration at the seven lake sites, with the model fitted separately for each site.

Team members: Abigail S.L. Lewis, Caleb J. Robbins

Archive directory: Forecast_submissions/Generate_forecasts/tg_lasso

XGBoost (xgboost_parallel)

The XGBoost model is an extreme gradient boosted random forest (XGBoost) machine learning model that uses predicted atmospheric conditions and day of year as covariates. This model utilises the *xgboost* R package (Chen & Guestrin 2016; Chen et al., 2023).

A new model was trained for each site daily using air temperature, solar radiation (surface downwelling shortwave flux in air), relative humidity, and day of year. Models were trained on a random sample of 80% of the historic data, reserving 20% for evaluation. Models have 15 trees and for each tree to have a maximum depth of 10. The model was then evaluated on the remaining samples, the error variance being recorded. A forecast with 31 ensemble members was generated for each day in the forecasting horizon using the ensemble members from the NOAA GEFS weather forecast, representing driver uncertainty. Those predictions then have normally distributed random noise added to them matching the recorded error variance. Model uncertainty is derived from NOAA ensemble members as well as random noise based on estimated model accuracy (process uncertainty).

This model was used to forecast water temperature and dissolved oxygen concentration at the seven lake sites, with the model fitted separately for each site.

Team members: Gregory Harrison, R. Quinn Thomas

Archive directory: XGBoosstNeon4Casts-main

References

Blanchard, G. F., Guarini, J. M., Richard, P., Gros, P., & Mornet, F. (1996). Quantifying the short-term temperature effect on light- saturated photosynthesis of intertidal microphytobenthos. Marine Ecology Progress Series, 134(1–3), 309–313. https://doi.org/10.3354/meps134309

Chapra, S.C. (2008). Surface Water-Quality Modeling. Waveland Press, Inc.

Chen, T., & Guestrin, C. (2016). XGBoost: A scalable Tree Boosting System. In Proceedings of the 22nd ACM SIGKDD International Conference on Knowledge Discovery and Data Mining (pp. 785–794). New York, NY, USA: ACM. https://doi.org/10.1145/2939672.2939785

Chen, T., He, T., Benesty, M., Khotilovich, V., Tang, Y., Cho, H., et al. (2023). xgboost: Extreme Gradient Boosting. R Package Version 1.7.5.1. Retrieved from https://cran.r-project.org/package=xgboost

Eppley, R. W. (1972). Temperature and phytoplankton growth in the sea. Fishery Bulletin, 70(4), 1063–1085.

Evensen, G. (2003). The Ensemble Kalman Filter: theoretical formulation and practical implementation. Ocean Dynamics, 53(4), 343–367. https://doi.org/10.1007/s10236-003-0036-9

Goudsmit, G.H., Burchard, H., Peeters, F., & Wüest, A. (2002). Application of k-ε turbulence models to enclosed basins: The role of internal seiches. Journal of Geophysical Research, 107(C12), 23-1–23-13. https://doi.org/10.1029/2001JC000954

Hamill, T. M., Whitaker, J. S., Shlyaeva, A., Bates, G., Fredrick, S., Pegion, P., et al. (2022). The Reanalysis for the Global Ensemble Forecast System, Version 12. Monthly Weather Review, 150(1), 59–79. https://doi.org/10.1175/MWR-D-21-0023.1

Herzen, J., Lässig, F., Piazzetta, S. G., Neuer, T., Tafti, L., Raille, G., et al. (2022). Darts: User-Friendly Modern Machine Learning for Time Series. Journal of Machine Learning Research, 23(124), 1–6. Retrieved from http://jmlr.org/papers/v23/21-1177.html

Hinshelwood C. N. (1945) The chemical kinetics of bacterial cells. Clarendon Press, Oxford

Hipsey, M. R., Bruce, L. C., Boon, C., Busch, B., Carey, C. C., Hamilton, D. P., et al. (2019). A General Lake Model (GLM 3.0) for linking with high-frequency sensor data from the Global Lake Ecological Observatory Network (GLEON). Geoscientific Model Development, 12(1), 473–523. https://doi.org/10.5194/gmd-12-473-2019

Hyndman, R. J., & Khandakar, Y. (2008). Automatic Time Series Forecasting: The forecast Package for R. Journal of Statistical Software, 27(3). https://doi.org/10.18637/jss.v027.i03

Hyndman, R. J., Athanasopoulos, G., Bergmeir, C., Caceres, G., Chhay, L., O'Hare-Wild, M., et al. (2023). forecast: Forecasting functions for time series and linear models. R package version 8.21.1. Retrieved from https://pkg.robjhyndman.com/forecast/

Kuhn M & Wickham H (2020). Tidymodels: a collection of packages for modeling and machine learning using tidyverse principles, https://www.tidymodels.org

Livingstone, D. M., & Imboden, D. M. (1989). Annual heat balance and equilibrium temperature of Lake Aegeri, Switzerland. Aquatic Sciences, 51(4), 351–369. https://doi.org/10.1007/BF00877177

Martin, J.L. and McCutcheon, S. (1998). Hydrodynamics and Transport for Water Quality Modeling. CRC Press

Monod, J. 1950. "Technique, Theory and Applications of Continuous Culture." Annales de l'Institut Pasteur 79 (4): 390–410.

Moore, T. N., Mesman, J. P., Ladwig, R., Feldbauer, J., Olsson, F., Pilla, R. M., et al. (2021). LakeEnsemblR: An R package that facilitates ensemble modelling of lakes. Environmental Modelling & Software, 143, 105101. https://doi.org/10.1016/j.envsoft.2021.105101

Moore, T. N., Mesman, J. P., Ladwig, R., Feldbauer, J., Olsson, F., Pilla, R. M., et al. (2021). LakeEnsemblR: An R package that facilitates ensemble modelling of lakes. Environmental Modelling & Software, 143, 105101. https://doi.org/10.1016/j.envsoft.2021.105101

Moulton TL (2018). rMR: Importing Data from Loligo Systems Software, Calculating Metabolic Rates and Critical Tensions. R package version 1.1.0, https://CRAN.R-project.org/package=rMR

Norberg, J. (2004). Biodiversity and ecosystem functioning: A complex adaptive systems approach. Limnology and Oceanography, 49, 1269–1277. https://doi.org/10.4319/lo.2004.49.4_part_2.1269

O'Hara-Wild, M., Hyndman, R., & Wang, E. (2022). fable: Forecasting Models for Tidy Time Series. R package version 0.3.2. Retrieved from https://cran.r-project.org/package=fable

Olsson, F., Carey, C. C., Boettiger, C., Harrison, G., Ladwig, R., Lapeyrolerie, M., Lewis, A. S. L., Montetealegre-Mora, F., Rabaey, J. S., Robbins, C. J., Yang, X., & Thomas, R. Q. (2024). What can we learn from 100,000 freshwater forecasts? A synthesis from the NEON Ecological Forecasting Challenge: model archive. Zenodo. https://doi.org/10.5281/zenodo.13750779

Rosso, L., Lobry, J. R., & Flandrois, J. P. (1993). An Unexpected Correlation between Cardinal Temperatures of Microbial Growth Highlighted by a New Model. Journal of Theoretical Biology, 162(4), 447–463. https://doi.org/10.1006/jtbi.1993.1099

Steele, J. H. (1962). Environmental Control of Photosynthesis in the Sea. Limnology and Oceanography, 7(2), 137–150. https://doi.org/10.4319/lo.1962.7.2.0137

Tay, J. K., Narasimhan, B., & Hastie, T. (2023). Elastic Net Regularization Paths for All Generalized Linear Models. Journal of Statistical Software, 106(1). https://doi.org/10.18637/jss.v106.i01

Taylor, S. J., & Letham, B. (2018). Forecasting at Scale. The American Statistician, 72(1), 37–45. https://doi.org/10.1080/00031305.2017.1380080

Thomas, R. Q., Figueiredo, R. J., Daneshmand, V., Bookout, B. J., Puckett, L. K., & Carey, C. C. (2020). A Near-Term Iterative Forecasting System Successfully Predicts Reservoir Hydrodynamics and Partitions Uncertainty in Real Time. Water Resources Research, 56, e2019WR026138. https://doi.org/10.1029/2019WR026138

Thomas, R. Q., McClure, R. P., Moore, T. N., Woelmer, W. M., Boettiger, C., Figueiredo, R. J., et al. (2023). Near-term forecasts of NEON lakes reveal gradients of environmental predictability across the US. Frontiers in Ecology and the Environment, 21(5), 220–226. https://doi.org/10.1002/fee.2623

Umlauf, L., Burchard, H., & Bolding, K. (2005). GOTM - Sourcecode and Test Case Documentation. Retrieved from http://www.gotm.net/pages/documentation/manual/stable/pdf/a4.pdf

Verburg, P., & Antenucci, J. P. (2010). Persistent unstable atmospheric boundary layer enhances sensible and latent heat loss in a tropical great lake: Lake Tanganyika. Journal of Geophysical Research, 115(D11), D11109. https://doi.org/10.1029/2009JD012839

Wright, M. N., & Ziegler, A. (2017). ranger: A Fast Implementation of Random Forests for High Dimensional Data in C++ and R. Journal of Statistical Software, 77(1). https://doi.org/10.18637/jss.v077.i01
